# The making of a (dog) movie star: The effect of the portrayal of dogs in movies on breed registrations in the United States

**DOI:** 10.1371/journal.pone.0261916

**Published:** 2022-01-12

**Authors:** Sarah Weir, Sharon E. Kessler

**Affiliations:** Department of Psychology, Faculty of Natural Sciences, University of Stirling, Stirling, Scotland, United Kingdom; University of Lincoln, UNITED KINGDOM

## Abstract

The media is a powerful force that can affect the welfare of the domiciled dog population. Dogs have long been in human stories and their depictions can create demand for the breeds shown. While previous research has found that this effect can last for up to ten years after the release of a movie, how this phenomenon occurs is unknown. This paper examines if how a dog is portrayed in a movie is associated with a subsequent change in American Kennel Club breed registrations for that breed. Following a systematic literature review, four key themes were identified in how dogs are portrayed in the media; dogs portrayed as heroes, as anthropomorphised, as embodying the ideals of Western societies (Whiteness and heteronormativity) and as boundaries between wilderness and human society. Forty movies from between 1930 to 2004 were analysed, resulting in 95 dog characters scored, and hierarchical multiple linear regression was run. Movies with dogs portrayed as heroes were followed by significant increases in the number of American Kennel Club breed registrations for the breed shown, while anthropomorphised dogs were followed by significant decreases in the number of dogs registered for up to five years after a movie’s release. These results indicate that how dogs are portrayed may be an important driver of demand for breeds. Future work should investigate whether these portrayals may have negative welfare implications for real dogs by leading to owners having unrealistic expectations for dogs or increasing demand for dogs with in-breeding related disorders.

## Introduction

Dogs have been used in human stories for centuries, usually to reflect human fears and anxieties [1]. A recent iteration of these stories is told through movies, and dogs have been critical to the medium’s development and popularity [2, 3]. From the very earliest movies, dogs have been central to the plot and were generally perceived as actors in their own right [4]. For example, the stars of the 1920’s, German Shepherds Strongheart and Rin Tin Tin, are even credited with almost single-handedly saving the Hollywood studios from financial collapse [2], showing that dog movies are immensely popular with the public and are therefore important media in our societies.

The popularity of dog movies suggests that they may influence the demand for the dogs shown. Multiple reports indicate that movies may create surges in purchases of particular animals [5–7], but we don’t yet know what it is about the portrayals that creates these surges. Dogs provide a unique case to better study this phenomenon because they have been frequently used in film from its creation and breed data exists. Using this data, Ghirlanda, Acerbi [8] found that breed popularity often increased after the release of a movie featuring a dog. However, it is unclear why some breeds became immensely popular after a movie (for example American Kennel Club (AKC) Irish Setters increased by 1500% after the release of Big Red in 1962), while other movies had no impact.

Despite dogs being critical to the development and success of cinema and the importance of portrayals in shaping public perception, dogs’ portrayals have been relatively unexplored [9]. Armbruster [1] believed that humanity scholars tended to see dogs as representations of human allegories, as disposable, frivolous objects, and not as a topic of legitimate study in their own right. Much of the published literature regarding dogs in human stories has been from the humanities, animal studies, literature and philosophy. To date, there has not been a systematic review to understand the different and common ways dogs have been portrayed in movies.

Following a systematic review (methods of which are detailed in the Methods section), four main themes have been identified; dogs as heroes (1), as anthropomorphised (2), portraying Whiteness and heteronormativity (referred to as Western ideals) (3) and as a boundary between wildness and human society (4).

### Dogs portrayed as heroes

The dog hero has been critical to the success of cinema [3, 10, 11]. Extremely popular dogs like Blair, the Border Collie, whose first film was in 1903 and Jean ‘The Vitagraph Dog’ who featured in films until her death in 1916, were important precursors to the dog hero by helping their families survive hardship or danger and help them earn a living [2]. However, it is “Teddy the Wonder Dog” who is believed to be the first dog depicted as a hero in the sense much of society would understand the term today [3]. Teddy, a Great Dane mix, rescued kidnapped babies and female leads, performed incredible stunts such as ‘driving’ a boat, and is described as incredibly intelligent by reviewers at the time [3]. Another famous dog Luke, the Staffordshire Bull Terrier, popularised dogs doing increasingly difficult stunts, like climbing ladders and being swung from the top of buildings [3]. At the time, dogs were becoming increasingly popular as sentimental family pets and so dogs like Teddy and Luke were used to widen the appeal of slapstick comedies to the whole family, resulting in the creation of the ‘family’ movie genre [3, 12].

It was the dogs in the 1920’s however, that cemented the tradition of the dog hero in film [2]. These dogs were depicted as brave, strong, loyal, and affectionate and always saved the day. They show no sign of fear and always values human life above its own [13]. While earlier dogs like Teddy would disappear for most of the movie to only reappear at the end to save the leading actress, Strongheart and Rin Tin Tin were the main protagonists [2, 3]. These dogs were valued and loved for setting an example for humans to return to the morals of simplicity, goodness and happiness [9]. Hero dogs were frequently depicted as ideal members of society and these depictions reflected what that ideal was in society at the time [14]. For example, Rin Tin Tin and Strongheart reflected the bravery and longing for clear cut morality during the 1^st^ World War, while Lassie represented the traditional values of loyalty and working-class pride in response to the industrialisation and modernity taking place during and after the 2^nd^ World War [2, 14]. This model of heroism continues to today, with protagonists such as *Balto* (1995) and *Hero Dog*: *The Journey Home* (2021) portraying many of the same traits as the original dog movie stars [15].

### Anthropomorphised dogs

This construction of dogs as ideal citizens also involved anthropomorphising them. Anthropomorphism is ascribing human or human-like qualities to non-human things [16]. It was common for the dogs in cinema to be spoken of by the press as ‘talented actors’ and many even received a wage [2, 3]. This humanisation of animals has been commonplace in stories for centuries (i.e. fairy tales such as Little Red Riding Hood), and the practice was easily transferred to film. Silent film enabled dogs to be placed on equal footing with their human co-stars and their performances were usually described and reviewed in the same way [2, 3]. Reviewers at the time noted the incredible acting ability of the dog performers to portray human-like emotion and on their ability to connect with viewers. Coren [17] notes that the early dogs on screen made the audience believe dogs in general could think, plan acts of retribution, remember complex facts and communicate easily with human language, because ‘hadn’t we actually seen Lassie do it?’

There are some common ways dogs are anthropomorphised in movies and literature. Many of the anthropomorphised dogs love humans unconditionally, understand them completely, can read and spell, and are depicted as low maintenance, obedient pets [18–20]. The more anthropomorphised a dog is, which is usually those digitally created and enhanced, the more appealing they are believed to be because their expressions are better suited to human stories [21]. Talking dogs are also well suited to movies where scripts and images are used to drive the narrative but they tend to voice strictly human perspectives [22]. Armbruster [22] discusses the danger of speaking for others, even when done with good intentions, because their experiences can be easily misrepresented, and important differences can be erased. Others believe, however, that it is this erasure of difference that can promote empathy for animals and that humans prefer animals that we can easily empathise with [22].

### Dogs portrayed as the ideals of Western societies

The idea that dogs on screen and in literature often represent the ideal values in society permeated much of the literature reviewed. While non-breed dogs came to represent social progress and working-class values, pedigree dogs were often described as upholding middle-class, White and heteronormative (the idea that promotes gender conformity, heterosexuality and family traditionalism as the norm and only way to be [23]) values as ideal in film [19].

From some of the first films, dogs were used to reinforce heteronormative standards by depicting a traditional family unit without the connotations of sex and childbirth [24–26]. Many of the early dog movie stars were used as surrogate children to a couple who was either too young, unmarried, or was too early in their relationship to have real ones. Examples of this narrative device include Teddy the Wonder Dog who starred in films in the 1910s, Asta in the 1930s, Pongo and Perdita’s romance in *One Hundred and One Dalmatians* in the 1960s and Marley in *Marley and Me* in the 2000s [3, 20, 24, 27]. All of these examples involve dogs working as surrogate children for the couple audiences are encouraged to root for and identify with.

Similar devices have been frequently employed to use dogs as a way to transmit ideas and assumptions about race [28]. Due to their real historical use to uphold colonial and eugenic hierarchies, dogs are especially well placed to transmit these assumptions [28–31]. A few movies deal directly with this history through their dog protagonists. For example, *White Dog* (1982), explicitly deals with the way dogs were used as tools to capture runaway slaves by trying to ‘deprogram’ a dog that was trained to attack Black people [31]. Most films, however, feature specific dog breeds that subtly transmit assumptions about race. For example, Labradors and Golden Retrievers are so often represented in the media as part of a white, nuclear, middle-class family, that they can now be counted upon to ‘safely transmit assumptions about whiteness’ [28]. Chihuahuas (pre Paris Hilton in the 1990’s [32]) were frequently used to depict negative Mexican stereotypes, such as Tito from *Oliver and Company* (1988) who starts fights, is promiscuous and hot-wires cars [33]. Rosenberg [28] believes that this use of dogs became especially prominent after the Civil Rights era in the 1960s, where public discourse about race in the United States became highly coded and hidden. Dogs in this environment became ‘a convenient wrapper for subtle racial messages and associations’ [28].

### Dogs portrayed as boundaries between wilderness and human society

The longest running theme identified was that dogs are portrayed as the boundary between wildness and human society. The story frequently begins with a wild dog joining a family and must conform to the accepted behaviours of humans [1]. This acceptance is usually achieved by demonstrating its devotion to its new family by shedding its wildness, usually through saving them from a wild animal [1]. Superle [34] describes these dogs as ‘benevolent helpers that straddle the distance between binary oppositions.’ This formula has been extensively used in literature for centuries but became especially popular in the 19^th^ Century when urbanisation was quickly becoming the norm [34, 35].

Cinema’s version of this narrative is commonly set in Antarctica, where native animals are scarce during winter and non-existent in the interior [36]. Sled dogs, whose breeds look wolfish and wild, are the only form of nature which form an uneasy bridge for humans to access wilderness [36]. The resulting fears and anxieties over human’s ability to dominate nature made these stories especially appealing and sometimes scary. For example, in John Carpenter’s *The Thing* (1982), the seemingly friendly husky dog at the beginning of the movie is actually an alien that absorbs everything in its path. In these narratives, dogs are boundary blurring, both representing the ‘civilised’ world and wildness. As they are able to form a bridge between these two realms, they are well placed to interrogate human’s deep cultural anxiety about establishing superiority over the natural world [1, 34, 37].

### A portrayal’s effect on breed registrations

Ghirlanda, Acerbi [8] has shown that movies portraying pedigreed dogs are sometimes followed by an increase in breed registrations for that particular breed, suggesting that increases in demand of dog breeds may be associated with movies. This paper builds on Ghirlanda, Acerbi [8], by specifically testing what aspects of the portrayals of pedigreed dogs are linked with increases in breed registrations. This paper tests for associations between the four key themes discussed above (dog heroes, anthropomorphism, Western ideals, and dogs as boundaries between wilderness and society) and changes in AKC breed registrations in the United States between 1930 and 2004.

## Methods

### Measuring a dog’s portrayal in a movie

To create a measure of a dog’s portrayal in human stories, a systematic literature review was conducted. Searches in Google Scholar and the University of Stirling Library Collection were conducted in January 2020. The search terms “dogs in literature”, “animals in literature”, “dogs in film”, “animals in film”, “canines in film”, “dog in movies” and “animals in movies” were used. There were no date restrictions and all material from English language journals or books were included. To reduce bias and ensure the widest range of papers were included, no outcomes were predetermined. Papers’ titles and abstracts were first scanned and any that did not mention dogs or animal stories were excluded. The remaining papers were then read, and key points were extracted that concerned how a dog was portrayed, how a fictional dog character was used in the story or what the dogs were used to represent. Any papers that did not discuss a dog’s portrayal were excluded. These key points were named based on their content in an iterative process. As new papers were read, key points were added to these categories and new ones were created if the existing categories did not represent them. This process resulted in ten categories. Many of these categories overlapped each other and so were collapsed into each other resulting in four themes. These formed the basis of the hypothesises used to determine if these ideas about how dogs are used in movies affected changes in breed registrations over time. See S1 File for a repeat review which followed the PRISMA guidelines, more detail about the searches conducted and the themes and sub-themes identified. The repeat PRISMA review retrieved papers that produced the same themes, confirming our original search (see S1 Appendix for the PRISMA checklist and flowchart). This process created the hypothesises below:

Hypothesis 1: Dogs portrayed as heroes will increase the respective breed registrations after the release of a movie compared to changes in all dog registrations over the same period (referred to as Dog Hero).
Hypothesis 2: Dogs that are anthropomorphised will increase the respective breed registrations after the release of a movie compared to changes in all dog registrations over the same period (referred to as Anthropomorphism).
Hypothesis 3: Dogs used to portray Western ideals will increase the respective breed registrations after the release of a movie compared to changes in all dog registrations over the same period (referred to as Western Ideals).
Hypothesis 4: Dogs portrayed as the boundary between wildness and human society (referred to Nature/Society Boundary) will increase the respective breed registrations after the release of a movie compared to changes in all dog registrations over the same period.

Movies produced and released in the United States between 1930 and 2004 were analysed. Those that featured at least one pedigree dog recognised by the AKC and were available on popular streaming sites to stream or rent were considered. (Please note, this paper uses the breed standards as defined by the AKC). Lists of dog movies were found online, and special attention was paid to the number of movies per decade. This was to ensure the sample of movies would not be biased towards a particular time period. All the Dogs list from IMDB was selected because the overall list had the largest number of movies from all decades included and other movies from Ghirlanda, Acerbi [8] not included in this list were subsequently added to enable comparison. Once the inclusion criteria were applied to the list, a total of 69 movies remained. Around five movies from each decade were analysed to enable the study to assess movies throughout the century. As there were 4 movies in the 1970s and 5 movies in the 1980s in the 69 movie sample, all movies from these decades were analysed. *After the Thin Man* (1936) and *Lassie Come Home* (1943) were selected because these were extremely popular movies at their time [14, 20]. All other movies were selected by stratified random sampling by decade. Movies were selected randomly until a total of 40 movies were analysed.

Included movies were made up of originals (movies that had not been made previously), sequels (a movie that continues the story, or expands upon earlier work), remakes (movies that are based on an earlier production) and re-releases (original movies that are released in theatres at a later point in time). Sequels were included in all analyses because the same characters can be portrayed differently in subsequent films, and so may have a different effect. Wolf [14] noted each movie of the Lassie franchise focused on different themes, and so the dogs were portrayed in different ways to fit these narratives. Remakes were included because they were deemed to be different enough from the originals. For example, characters would sometimes be changed, like in the 1996 remake of *One Hundred and One Dalmatians* where the Old English Sheepdog ‘Colonel’ was changed into an Airedale called ‘Kipper’. Finally, rereleases were included because although the movie and characters were the same, the portrayals will be compared against different data at a later point in time. However, as the content was exactly the same and therefore the portrayal of the dog was the same, there was a risk that the assumption of independence could be broken. Therefore, statistical tests were run with rereleases both included and excluded and if results were different, rereleases would be excluded.

To measure the degree to which dogs were portraying the four themes identified, a quantitative content analysis approach was used [38]. Criteria were developed to operationalise the four conceptual themes and allow the dog characters’ portrayals to be systematically coded. The criteria were developed using the same papers identified for the literature review (See S1 File for details of the literature review and S1 Table for scoring criteria). Many of these essays deconstructed the conceptual themes and these discussions were used as the basis of the scoring system.

Pedigree dog characters recognised by the AKC who were on screen for 5 minutes or longer were included and were identified while analysing the full movies. Characters were scored a 1 if the criteria were identified or a 0 if not. A pilot study of the first five movies was conducted to test and refine the coding criteria. These movies were coded again during the main study. See S1 Table for criteria developed and the source they came from. Notes were taken for each score, detailing what the character did to receive a 1 or 0 which can be found in S2 File.

After watching each movie, the scores for each character were automatically summed in Excel and a percentage of the total number of criteria was created. For example, Toto from The Wizard of Oz was observed performing 4 of the 17 criteria regarding the ‘hero hypothesis’ and so received a score of 19%. See S2 File for the list of movies and characters included in the study, the scoring given for each character and final data used in the study.

### Measuring dog breed changes

With each character’s portrayal scored, the aim was to determine whether this influenced breed registrations. The AKC has the largest and longest running pedigree registry in the world. Each year, the number of new puppies registered with the AKC are published, with data up to 2005 freely available online [39]. Although not all purebred dogs are registered with the AKC and not all dogs are AKC purebreds, the large number of dogs registered between 1930 and 2005 (N = 65,119,362) provide a reasonably accurate account of the relative popularity of breeds throughout the 20^th^ Century [40]. With this large dataset, AKC breed registrations were used as a proxy measure of buying behaviour for dog breeds.

The effect of a character on the subsequent breed registrations was determined by using an index of change developed by Ghirlanda, Acerbi [8]. The aim was to ensure any increases found were a result of the movie in question and not because the breed was already becoming more popular before its release.

The index is calculated by:

$$T_{n}=100\times \frac{a_{n}-b_{n}}{p_{n}}$$

Where a_n_ is the average change in registrations from the release of the movie to n years after movie release (and where n is the number of years after a movie being analysed). b_n_ is the average change in registrations from n years before the movie release to the release of the movie. p_n_ is the average dog breed registrations over the course of the period.

Ghirlanda, Acerbi [8] reported that the long-term changes in breed registrations declined over time, with all characters in the 1990’s experiencing negative changes. On further inspection, the total number of AKC registrations fluctuates throughout the 20^th^ Century. There are large increases in the 1960s and sharp declines in the 1970s and 1990s. These fluctuations are likely due to either changes in attitudes towards dog ownership as a whole or attitudes towards purebred dogs [41]. To account for these fluctuations, Ghirlanda et al.’s index of change was modified. In this paper, the breed index of change was compared to the index of change for the overall dog registrations for the period being investigated. Therefore, if all breed registrations are declining but one breed declines at a decreased rate due to a release of a movie, an effect can still be found.

Y=indexofchangeofdogbreed−indexofchangeofalldogbreeds

Using this method, 1, 2, 5- and 10-year index of changes were conducted to be comparable to Ghirlanda, Acerbi [8] while also controlling for the broader changes in dog ownership occurring in society during the 20^th^ Century. These periods may indicate how the various portrayals will affect different motivations for purchases. Shorter term changes may highlight viewers who bought dogs almost immediately after watching a movie, suggesting an impulse buy. Five- and ten-year changes may suggest that a dog influenced viewers’ perceptions of what an ideal dog looks like, and so affected their longer-term purchasing behaviour.

As breed data is only available between 1926 and 2005, five-year changes will not be calculated for movies released before 1931 or after 2000 and ten-year changes will not be calculated before 1936 or after 1996.

### Statistical analysis

#### Hypothesis testing

The assumptions of a multiple linear regression model were met, and results of tests are included in S2 Appendix. A model building approach was conducted using a hierarchical stepwise method, where predictors are selected based on previous work and are added one at a time depending on their importance in existing literature [42]. The importance of a theme in cinema history, the prevalence of its discussion in the literature and how often the theme was featured in the study’s sample were used to assess the order of predictors. The importance of a theme in cinema history was given priority because it was expected that if a theme was critical to the development of the popularity of cinema and in the development of genres, it would have the greatest impact on the audiences at the time of a movie release (see Table 1). After ranking the predictors, Dog Hero was entered first, Anthropomorphism second, Western Ideals third and Nature/Society Boundary fourth.

**Table 1:** Method of determining the order of predictors in model.

Hypotheses	Importance in cinema history^1^	Percent of dogs who performed at least 50% of hypothesises criteria^2^	Percent of sources that discuss themes^3^	Avg Score^4^	Order of Model Entry
Dog Hero	8	3	2	4.33	1
Anthropomorphism	6	2	4	4.00	2
Western Ideals	4	4	3	3.67	3
Nature/Society Boundary	2	1	1	1.33	4

*Note. ^1^The importance in cinema history score was determined by the content of sources and the importance that they ascribed to these depictions in cinema history. To prioritise the hypothesises role in cinema history, a score of 1–4 (1 being least important and 4 being most) was doubled.

^2^Determined after scoring the included characters from the movie sample. Scores were in percentages of dogs in sample that performed at least 50% of the hypothesis’s behaviours. These were ranked from 1–4, with 4 being the hypothesis that the most dogs were portraying and 1 being the least.

^3^For a source to be determined as discussing a theme, it needed to be mentioned at least once in a paper. A source could mention more than 1 theme. These mentions were summed, and a percentage of the 23 total sources was created. These were ranked from 1–4 (with 4 being awarded to the hypothesis that was discussed most frequently and 1 being the least frequently discussed).

^4^Avg Score is determined by calculating the average of the three criteria.

Dog heroes were determined to be the most important in film history because it was credited with the creation and development of the ‘family’ genre [3]. Dogs were used to widen the appeal of slapstick or “knockabout” comedy from working class men to the entire family in the 1910’s [3]. Dog heroes became even more key when the Hay’s Codes, Hollywood’s self-censorship as a result of pressure from the Catholic Church, came into force because they were used as a child substitute for grown up sexual couples who’s sex lives could not be explicitly discussed [3, 43]. A number of researchers such as Rapf [3], Fuller-Seeley and Groskopf [2] and Wolf [14] focused their essays entirely on the dog hero and its contribution to film history and the importance of this depiction in movies. Therefore, although only 12 sources of the 30 papers mentioned dog heroes at least once (compared to 18 sources for anthropomorphism and 14 sources for Western ideals), the importance and prevalence of the theme was emphasised.

In contrast, few if any researchers focused exclusively on anthropomorphism and Western ideals and their importance in cinema development (For example, only Rosenberg [28] and Quinn [24] focused solely on Western ideals). Greater focus has been placed on the effects of intensifying the anthropomorphism of dogs in movies through the use of technology in more recent years and so Anthropomorphism was awarded a higher score than Western Ideals [18, 21]. Dogs portrayed as a boundary between wilderness and human society was awarded the lowest overall score for each criterion because although this theme was identified by researchers as important in literature, it featured little in discussions surrounding film. Table 1 outlines the scoring and the calculated order of predictors. Each model with an additional predictor was then tested using an ANOVA to determine if these additions significantly improved the model fit [42].

Using G*Power 3.1.9.4 post hoc power analysis was completed. Using Linear multiple regression: Fixed model, R^2^ deviation from zero with effect size as 0.15, 4 predictors and a sample size of 95 dogs, the achieved power was 85%. However, the achieved power was 83% for 5- year changes with a sample size of 90 dogs and 73% for 10-year changes with a sample size of 74 dogs.

#### Exploratory analysis

Additional exploratory analyses, using t-tests and regression models, were conducted to determine if the way a dog was portrayed was affected by the character’s sex, film type or the decade of movie release. The characters from the rereleased movies provided an interesting opportunity to see how the same movie affects breed registrations across time, and so these were tested individually.

All statistical tests were conducted in R Version 1.2.5042. Significance was determined at an alpha of p < 0.05.

### Ethical issues

No ethical issues were identified. The University of Stirling Ethics Checklist was completed, and it was determined that no further review was required because the study only involved collecting data from databases and movies.

## Results

### Distribution of scoring

Characters were more likely to score highly on the Dog Hero and Western Ideals hypothesises with over 80% of characters scoring at least 25% on both. There was usually at least a little anthropomorphism depicted with over 50% of characters scoring at least 25%. Dogs portrayed as the boundary between nature and human society were rarely depicted in the sample with only 14% of characters scoring at least 25%. See full breakdown of scoring in Table 2.

**Table 2:** Distribution of scores for each hypothesis.

Hypothesis	Score of at least 25%	Score of at least 50%	Score of at least 75%
Dog Hero	87%	54%	20%
Anthropomorphism	56%	21%	7%
Western Ideals	82%	62%	25%
Nature/Society Boundary	14%	6%	0%

*Note*. Scores exclude rereleases.

### Hypothesis testing

#### Identifying the optimal model

An ANOVA for linear models was used to determine which model was the best fit for the data. The ANOVA compared each model that had an additional predictor to determine if its addition significantly improved model fit [42]. Results indicated that model 2 which included scores for Dog Hero and Anthropomorphism, improved the model fit for 1 year changes, F(1,92) = 4.79, p = 0.031, 2 year changes, F(1,92) = 5.13, p = 0.026 and 5 year changes, F(1,87) = 5.60, p = 0.020. Adding Western Ideals and Nature/Human Society Boundary did not significantly improve the fit of the model and so the multiple regression was run using model 2 for these periods. Results for 10-year changes did not have any significant results and so adding more predictors did not improve the fit of the model. See Table 3 for results.

**Table 3:** ANOVA results to find the model that best fits the data.

Time Period	Model Number	Residual Degrees of Freedom	Residual Sum of Squares	Degrees of Freedom	Sum of Squares	F Statistic	p-value
1 Year Changes	Model 1	93	3943.3				
	**Model 2**	**92**	**3744.4**	**1**	**198.88**	**4.79**	0.031*
	Model 3	91	3738.9	1	5.51	0.13	0.717
	Model 4	90	3737.1	1	1.87	0.05	0.832
2 Year Changes	Model 1	93	8958.9				
	**Model 2**	**92**	**8481.6**	**1**	**477.34**	**5.13**	0.026*
	Model 3	91	8375.4	1	106.22	1.14	0.288
	Model 4	90	8373	1	2.34	0.03	0.874
5 Year Changes	Model 1	88	15997				
	**Model 2**	**87**	**15011**	**1**	**985.97**	**5.60**	0.020*
	Model 3	86	14984	1	27.23	0.15	0.695
	Model 4	85	14964	1	19.86	0.11	0.738
10 Year Changes	Model 1	72	17570				
	Model 2	71	17305	1	265.82	1.07	0.30
	Model 3	70	17109	1	196.00	0.79	0.38
	**Model 4**	**69**	**17101**	**1**	**7.16**	**0.03**	**0.87**

*Note*. Bold indicates selected model and * indicates p < 0 0.05.

Once it was determined that Model 2 was the best fit for periods 1, 2 and 5 years, multiple linear regression was run. As no results were significant for 10-year changes, Model 4 was run. To confirm the Hierarchical Stepwise a priori approach using an ANOVA to compare models was not biasing the results, AICs were calculated for the models created using a Stepwise model. A Best-Subsets approach was also run to ensure the a prior predictor order was not biasing results, and the resulting models were compared using a adjusted R^2^, CP’s Mallow and BIC criterion. These approaches confirmed the original findings (See S6 Appendix for results).

#### Multiple regression results

With Dog Hero and Anthropomorphism added as predictors, the model explained 10.31% of variance for breed registration changes one year after movie release (*F*(2,92) = 5.29, *p =* 0.007), 14.18% of variance for two years after movie release (*F*(2,92) = 7.60, *p =* 0.001) and 13.03% of variance for five years (*F*(2,87) = 6.52, *p =* 0.002). Dogs portrayed as heroes were significant, positively predicting the likelihood of increases in AKC registered dogs one year (*t* = 0.33, *p =* 0.003), two years (*t* = 0.40, *p <* 0.001) and five years (*t* = 0.37, *p =* 0.001) after the release of a movie compared to all registered dogs. Dogs that were anthropomorphised on the other hand were significant, negatively predicting likelihood of increases in AKC registered dogs one year (*t* = - 0.24, *p =* 0.030), two years (*t* = - 0.24, *p =* 0.030) and five years (*t* = - 0.26, *p =* 0.019) after movie release compared to all registered dogs.

None of the predictors significantly predicted changes in AKC dog breed registrations ten years after the release of a movie, *F*(4,69) = 1.027, *p =* 0.400 (Dogs heroes, *t* = 0.26, *p =* 0.058, Anthropomorphism, *t* = - 0.09, *p =* 0.510, Western Ideals, *t* = - 0.12, *p =* 0.376 and Nature/Society Boundary, *t* = - 0.02, *p =* 0.866), indicating that the dogs’ portrayals did not affect AKC registration changes.

### Characters analysed

**Fig 1:**
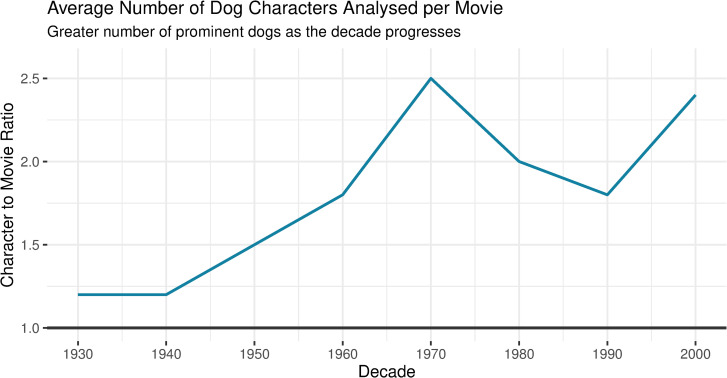
Average number of characters per movie by decade.

A total of 40 movies were examined, resulting in 71 characters analysed. The number of characters per movie significantly increased as the century went on (ANOVA, *F*(1, 6) = 11.0, *p <* 0.05), from 1.2 characters per movie during the 1930s to 2.4 characters in the 2000s. See Fig 1 for the average number of characters per movie in each decade analysed.

The average number of prominent dog characters in each decade significantly increased as the century progressed. A peak in 1970 is a result of six Dobermans in *The Doberman Gang (1972)*. To find the dog character to movie ratio, the total number of dog characters analysed each decade were divided by the total number of movies included in the sample. An ANOVA was used to determine whether the average number of dog characters significantly changed over time.

The multiple regression model was run with rereleases included and excluded. Both tests provided the same overall results and so results reported are with characters in rereleases included. See S3 Appendix for results of tests with rereleases excluded.

**Fig 2:**
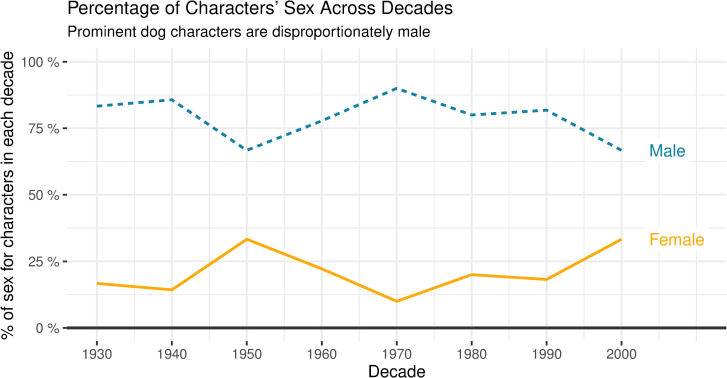
Percentage of the sex of characters across decades.

76% of the 95 characters included in the study were male (N = 72) and 24% were female (N = 23). The sex breakdown of the characters did not significantly change across decades (ANOVA, *F*(1, 93) = 0.40, *p >* 0.05). See Fig 2 for the percentage of the sex of characters across the time period analysed.

Male dogs were overrepresented in the sample where 76% of dog characters analysed were male. The percentage of the characters’ sex did not significantly change throughout the 20^th^ Century.

### Exploratory analysis

#### How sex of character affects portrayal

**Fig 3:**
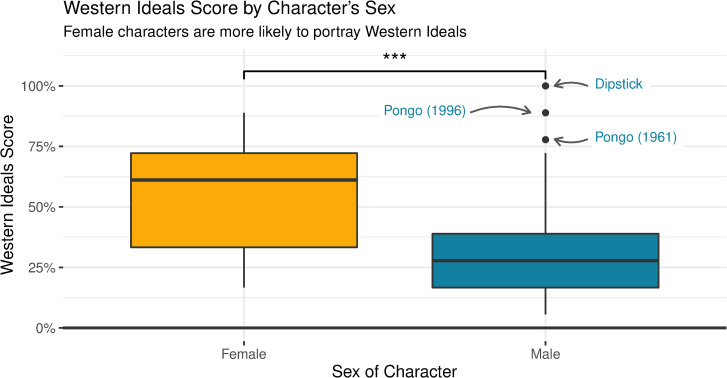
Western ideals score by sex of character.

Exploratory analysis was conducted to gain a better understanding of how different attributes of the character or movie affected a dog’s portrayal. The data was broken down by sex and was tested for normality. All normality tests for each sex were significant, *p <* 0.05, except for female characters’ scores for Dog Hero, *p =* 0.106. Therefore, differences between sex were calculated using Wilcox rank sum tests [42]. The sex of characters significantly affected the portrayal of Western values. Female characters portrayed Western values significantly more (*Median* = 0.61) than male characters (*Median* = 0.28), *W* = 1235.5, *p <* 0.001, *r* = - 0.36. See Fig 3 for a visual comparison. There was no significant difference between the scores male and female characters received for Dog Hero (Female (*Median* = 0.41), Male (*Median* = 0.50), *W* = 693, *p =* 0.240, *r* = - 0.12), Anthropomorphism (Female (*Median* = 0.67), Male (*Median* = 0.58), *W* = 926, *p =* 0.394, *r* = - 0.09) and Nature/Society Boundary (Female (*Median* = 0.00), Male (*Median* = 0.00), *W* = 862.5, *p =* 0.722, *r* = -0.04).

Female dogs analysed were significantly more likely to have a higher score for Western Ideals. There were 3 male characters who were outliers, all from the *One Hundred and One Dalmatians* franchise; Pongo from *One Hundred and One Dalmatians* (1961), Pongo from the 1991 remake and Dipstick from *102 Dalmatians* (2000).

#### How the type of movie affects portrayal

Wilcox rank sum tests were used to test if dogs were portrayed differently depending on whether the movie was live action or animated. These tests were used because normality tests were significant, p < 0.05 [42]. Characters featured in animation movies had significantly higher scores for Anthropomorphism (*Median* = 0.75) compared to those featured in live action films (*Median* = 0.50), *W* = 1642, *p <* 0.001, *r* = - 0.44. Characters featured in animation also tended to have higher scores for Western Ideals (*Median* = 0.33) compared to those featured in live action films (*Median* = 0.28), although this difference was not significant, *W* = 1338.5, *p =* 0.052, *r* = - 0.20. Characters featured in live action films tended to be scored significantly higher in Nature/Society Boundary (*Median* = 0.00) than those featured in animation (*Median* = 0.00), although the scores were generally very low, *W* = 780.5, *p =* 0.006, *r* = -0.28. There was no difference between dogs portrayed as heroes in live action (*Median* = 0.53) and animation movies (*Median* = 0.35), *W* = 896.5, *p =* 0.156, *r* = - 0.15.

#### How the portrayal changes across decades

The portrayal of the characters did not significantly change over time. Using ANOVAs, there was no significant change between decades in the scores of Dog Hero, *F*(1, 93) = 2.78, *p >* 0.05, Anthropomorphism, *F*(1, 93) = 2.61, *p >* 0.05, Western Ideals, *F*(1, 93) = 1.21, *p >* 0.05 and Nature/Society Boundary, *F*(1, 93) = 0.39, *p >* 0.05.

#### Breed registration changes of characters in rereleases over time

**Fig 4:**
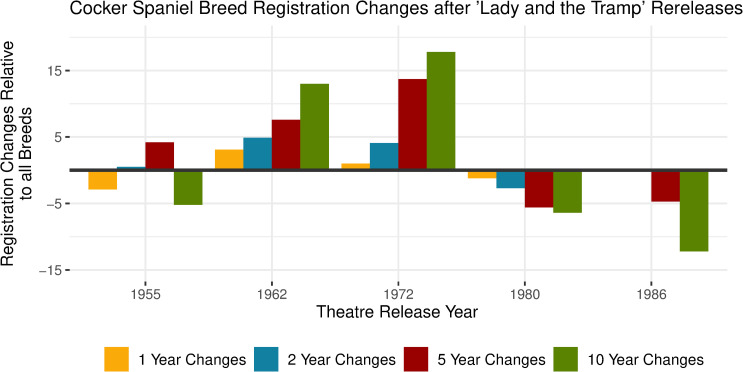
Cocker spaniel breed registration changes after ’Lady and the Tramp’ rereleases.

Changes in breed registrations were different after the additional showings of *Lady and the Tramp* (1955) and *One Hundred and One Dalmatians* (1961). After the rereleases of *Lady and the Tramp* (1955) in 1962 and 1972, there were large increases in Cocker Spaniel registrations compared to the overall dog registrations. In the 1980s, both releases were followed by decreases in changes in the Cocker Spaniel breed registrations. See Fig 4 for changes in registrations after the movie was rereleased.

*Lady and the Tramp* (1955) was released into theatres another 4 times after its original release in 1955; in 1962, 1972, 1980 and 1986. The popularity of Cocker Spaniels relative to all AKC registered dogs changed in different ways relative to all dog registrations depending on the year of release. Negative values indicate that the Cocker Spaniel registrations declined relative to the change of all dog registrations. The Box Office sales during the opening weekend adjusted for inflation do not account for these changes. See S2 Table for all inflation adjusted Box Office figures.

**Fig 5:**
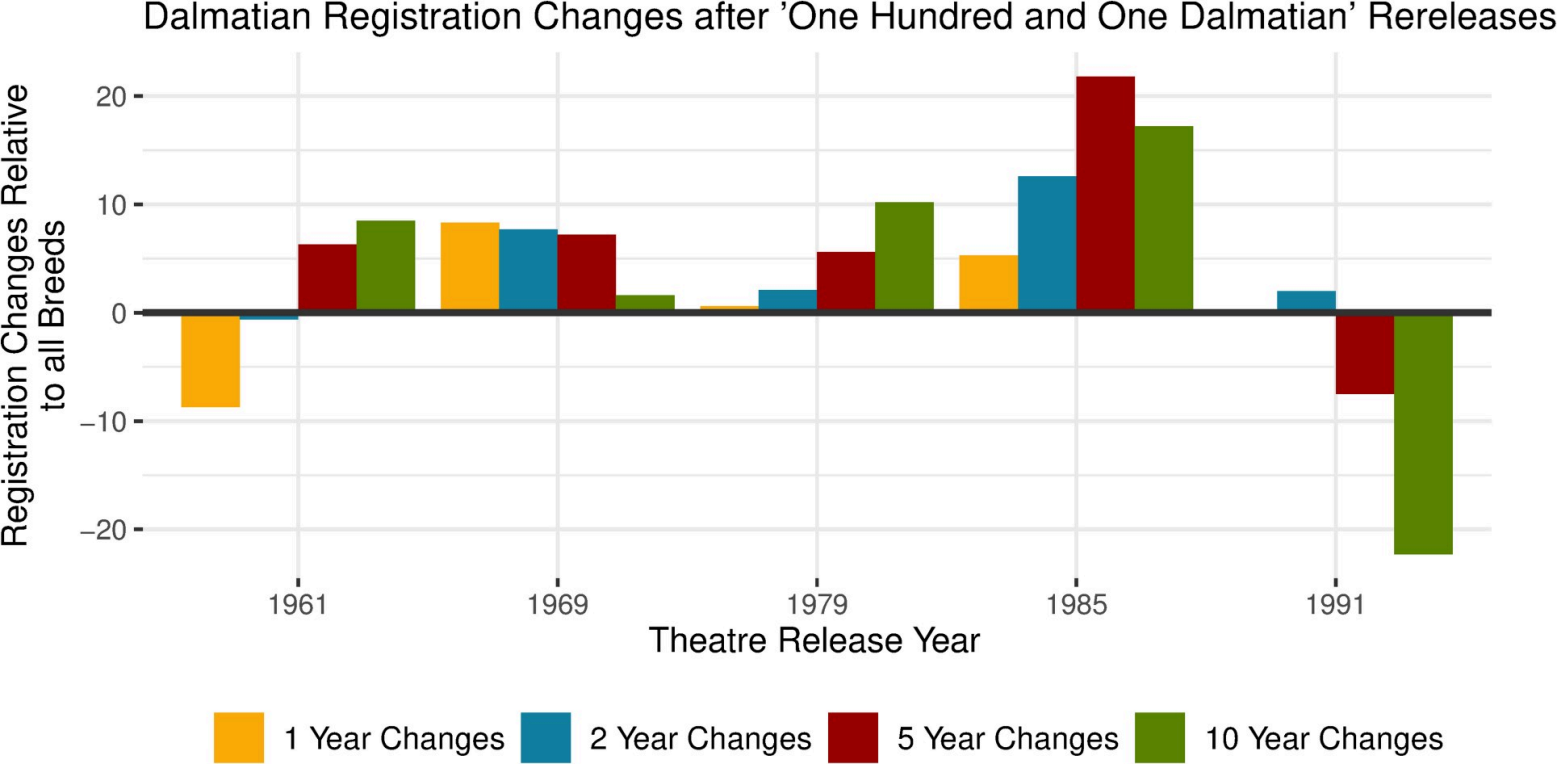
Dalmatian registration changes after ‘One Hundred and One Dalmatian’ rereleases.

The changes in Dalmatians registrations compared to the overall dog registrations were also different after *One Hundred and One Dalmatians* (1961) rereleases. Dalmatian registrations increased after the 1979 and 1985 rereleases and then declined after the 1991 release compared to all dog registrations. See Fig 5 for changes in registrations after each rerelease.

The original animated *One Hundred and One Dalmatians* (1961) was rereleased in theatres 4 different times throughout the United States after its original release in 1961; in 1969, 1979, 1985 and finally in 1991. These changes do not include the 1996 live action remake named *101 Dalmatians* (1996). Negative values indicate that the Dalmatians registrations declined relative to the change of all dog registrations. Again the changes in Dalmatian registrations did not align with Box Office sales adjusted for inflation during the opening weekends. See S2 Table for all Box Office figures.

## Discussion

The present study supports the idea that how a dog is portrayed in a movie can have a powerful impact on breed registrations. Significant changes were found to occur for up to 5 years after a movie was released, suggesting that the portrayal of dogs in movies impacts dog breed popularity. This study builds on prior work by Ghirlanda, Acerbi [8] in two ways. First, by identifying how specific themes in the movies influence registrations and second, by comparing changes in dog breed registrations after a movie’s release to the overall changes in all AKC registered dogs. This allowed the current study to analyse changes relative to overall changes occurring in the ownership of pedigreed dogs. And indeed, considering the huge societal changes that have occurred over the 20^th^ Century, the characters’ portrayals represented a relatively substantial amount of variance (10–15%) in the AKC registrations. This indicates that the way a dog is depicted in a movie may be an important driver in how prospective dog owners choose a breed and suggests there is a real effect occurring. This supports Ghirlanda, Acerbi [44]’s findings that dog owners tend to not base their decision on breed health, longevity or behavioural characteristics.

However, breed registrations did not significantly change in relation to all breed registrations after 5 years as was found in Ghirlanda, Acerbi [8]’s results. An explanation for this may be that the portrayal of a dog has only a short-term impact and there are other factors at play in longer term trends. Other factors such as the level of a movie’s promotion or how often that breed is featured in other movies around the time, may cause the registrations to continue to increase in the longer term. However, it may also be due to the loss in power of the study when investigating 10-year changes. As only breed data from 1926 to 2005 was available, the study could not test movies for 10-year changes if they were released before 1936 or after 1996. With the resulting loss of participants, it may have been more challenging to find a true effect.

### Dogs portrayed as heroes

Dogs portrayed as heroes were the only type of portrayal that significantly increased the number of dogs registered after the release of a movie. Dog heroes were the most frequently depicted across the decades. The results can shed light on what it means to be a ‘good dog’ in Western societies [44]. Many of the actions the majority of characters performed included showing allegiance and affection to their family or human. Many dog heroes saved their humans from death or if they couldn’t be saved, slept on their dead owners’ graves (like Bobby in *Greyfriars’s Bobby* (1961)) or even avenged their deaths (like Shep in *The Painted Hills* (1951)). In real life, these acts and stories are often rewarded. Wynne [45] notes Western society’s longstanding obsession with dogs saving humans. Animals are often awarded medals and OBEs after performing acts that are deemed heroic, and books written about their lives are featured in bestseller lists [46, 47].

Holding these actions as the ideal way dogs should behave is problematic. Bradshaw [47] worries that believing dog heroes consciously save people encourages the public to believe their pets think as humans do. It also romanticises behaviours that are distressing and dangerous for dogs. For example, multiple movies portrayed separation anxiety as a dog’s devotion to their owner, rather than a highly stressful state for the animal. In *Big Red* (1962) for example, Red jumps through a window almost killing himself after being extremely distressed at being separated from the boy who normally cares for him. Romanticising a common issue that is stressful for dogs (and humans who come home to destruction) may make owners less likely to successfully prepare their dogs to be left alone. It has been reported that the prevalence of separation anxiety in the US dog population is approximately 20% [48, 49]. Further study investigating the effects on the expectations of owners after watching a movie featuring a hero dog would be useful.

### Anthropomorphised dogs

Anthropomorphised dogs also created significant changes, but they significantly decreased the AKC dog registrations up to 5 years after a movie’s release compared with all dog registrations. This was unexpected as it was predicted that viewers would prefer humanised animals that were easier to empathise with and who were portrayed as unrealistically low maintenance pets [19, 22]. McLean [18] believed that humans enjoy movies with talking and anthropomorphised dogs because it validates the ‘hope and dream’ that dogs love us unconditionally. This study’s results may point to another more sinister reason why people may avoid purchasing a dog portrayed as anthropomorphic. Armbruster [22] expressed that anthropomorphised and talking dogs powerfully confirm our belief in human superiority by finding comedy in how imperfectly they attempt our actions. Instead of helping humans understand and empathise with dogs by making them more human, anthropomorphism may instead be highlighting dogs’ differences from humans. Their imperfection at the things that are supposedly human others them instead of creating a connection.

A less troubling reason for this result, although not mutually exclusive, is that the dogs who had higher scores on anthropomorphism were likely to be seen by more children than adults. Anthropomorphised dogs were significantly more likely to be portrayed in animated movies which were usually aimed at children rather than a more general audience. These results therefore may indicate that the audience may influence whether there are substantial changes in breed registrations. It may be that movies designed for children only create these decreases because the movie is not being watched by decision makers. It may also be that the children watching movies depicting certain dog breeds would only purchase that dog later as adults. As the emotional response of animal characters can be especially long lasting [50], images of dogs seen in children’s films may inadvertently influence dogs purchased later in life.

### Dogs portrayed as the ideals of western societies

Dogs portraying Western Ideals did not have a significant effect on people purchasing dogs which was also unexpected. It was predicted that viewers were more likely to imagine adding a dog to their family if they embodied mainstream values. A number of researchers referred to how dogs frequently represent ideal societal attitudes in the media, such as class, race, heteronormativity and gender roles [14, 19, 28]. However Wiersma [51] found that gender images in Disney movies did not evolve to match the changes occurring in society. Therefore, the ‘family’ movie may be more conservative than movies in general. The depiction of the dogs featured in the sample may not have aligned with the values of most viewers. Also, McHugh [19] found later in the 20^th^ Century, breed dogs began to represent the status quo, while mixed breed dogs began to symbolise progressive social change. This can be especially seen in the 1970’s where many of the most popular and acclaimed movies featured mixed breed dogs, such as *Benji* (1974) who became the biggest star of the decade. The exclusion of mixed breed dogs may have prevented an effective comparison of the effects of Western Ideals.

These results may also be due to how widespread these ideas were in the sample. Breed registrations may not be affected because there is little difference between depictions. Most of the characters had high Western Ideals scores. All but 2 dog characters (Tito and Rita in *Oliver and Company* (1988)) were portrayed as White and all the dogs belonged to White families except 3 (Moreover in *The Biscuit Eater* (1972) and Nana and Demon in *Snow Dogs* (2002)). Seventy-six percent of the dogs included in the study were male. The females included were significantly more likely to portray Western Ideals, likely because they were frequently mothers or love interests. All romantic relationships were straight and monogamous despite dogs being promiscuous (a male dog is estimated to sire over a hundred litters in his life and females come into season multiple times a year [52]). With very few differences in depiction, Western ideals may be unlikely to have an impact on viewers.

### Dogs portrayed as boundaries between wilderness and human society

The story of a stray or wild dog becoming civilised, crossing the boundary to join human society as reformed, was rarely depicted in movies despite being common in literature. Likely as a result, it did not affect a viewer’s purchasing decisions. Dogs portrayed in wild settings, like Western United States or Antarctica, that would be portrayed as a boundary between wilderness and human society in literature, would instead be portrayed as hero dogs [1, 36].

## Conclusion

The results show that portrayals of dogs as heroes in movies is associated with an increase in registrations of that breed, suggesting that the movies may be contributing to the demand for those breeds. This may have welfare implications for the dogs. If movie portrayals influence what the public expects from the dogs they purchase, owners who purchase dog hero breeds may have unrealistic expectations. This may be similar to what McHugh [19] described as the ‘misrepresentation of low-maintenance family pets’. Most dogs in the sample were extremely obedient, could communicate easily with people using human language or barking, showed affection to their family and none of their daily needs were shown. This could leave owners unprepared for the realities of dog ownership. Adding to this issue is that breeds frequently depicted as dog heroes, such as German Shepherds and Dalmatians, have more reported undesirable behaviours that are some of the top reasons for relinquishment [53, 54]. Armbruster [45] believes that while these depictions validate the existence of dogs that meet the heroic criteria, they also suggest that those who do not measure up should be considered disposable. Future research should investigate whether dog hero breeds are being relinquished and whether owners have different expectations of these breeds relative to other breeds. Media literacy and dog ownership education could also be provided during the promotion of dog movies, especially those featuring dog heroes, as these dog breeds may be particularly vulnerable to failing to live up to their owners’ subconscious expectations.

### Future directions

The methods used in this study could be adapted to better understand how villain dogs and mixed dog breeds influence demand for dogs. For example, *Cujo* (1983) was depicted as something to be fearful of, but there were no criteria in this study to capture this. Cujo’s scores were therefore extremely low but surprisingly, there were large increases of Saint Bernard’s puppy registrations after the release of the movie. Breaking down the targeted audience of movies may also be helpful to understand how this affects results.

In addition, this study included only dog breeds which are registered with the AKC at the time the movie was released. This limited the movies and dog characters that could be analysed. As Hollywood encodes messages in the breeds of dogs [27], dog breeds not recognised or mixed breed dogs may be portrayed differently from those included in the sample. Mixed breed or non-breed dogs became increasingly associated with social critique while the breed dog came to represent the status quo as the 20^th^ Century progressed [19]. Future research could analyse how breed and non-breed dogs are represented in film and explore how these representations affect adoptions from shelters.

## Supporting information

S1 File Literature review details and results. (XLSX)

S2 File The making of a (dog) movie star (data) also available here. (XLSX)

S1 Table Criteria to score characters. (XLSX)

S2 Table Adjusted box office figures for rereleases. (XLSX)

S1 Appendix PRISMA literature review flowchart and checklist. (DOCX)

S2 Appendix Assumption test results. (DOCX)

S3 Appendix Results with rereleases excluded. (DOCX)

S4 Appendix Movies cited. (DOCX)

S5 Appendix The making of a (dog) movie star (code). (TXT)

S6 Appendix Best subset method and AIC results. (DOCX)
