# Supplementary material for: The making of a (dog) movie star: The effect of the portrayal of dogs in movies on breed registrations in the United States
Source: PLoS One. 2022 Jan 12;17(1):e0261916. doi: 10.1371/journal.pone.0261916 (PMC8754329; doi:10.1371/journal.pone.0261916)
Supplement: S2 Appendix — (DOCX) [file pone.0261916.s006.docx]

## S2 Appendix. Assumption tests for the multiple linear regression models.

***Outliers and Influencers***

Standardised residuals were calculated to determine which data points were outliers. Data points were considered possible outliers when a character’s standardised residual was less than -2 or greater than 2 (Field, Miles, & Field, 2012). According to the normal distribution 95% of standardised scores should be within the range of -1.96 and +1.96. 99% of the scores should be within -3.29 and +3.29. According to these values, all characters with scores outside of +/- 2 were checked to determine if they would cause the model to be a poor fit to the data as well to ensure the data points were not errors.

Characters who had standardised residuals either greater than +2 or less than -2 for each change period were identified. The characters’ scores were then checked using average leverage, covariance and Cook’s Distance to determine if the level of error within the model was acceptable and if any of the scores were applying undue influence on the model.

One Year Changes

| Table 4 |  |  |  |  |  |  |  |
| --- | --- | --- | --- | --- | --- | --- | --- |
| *Characters whose Standardised Residuals were less than -2 or greater than +2* | | | | | | | |
| Movie | Year | Character | Breed | Standard - ised Residuals | Cooks Distance | Leverage | Covariance Ratios |
| Cats & Dogs | 2001 | Butch | Anatolian Shepherd | -3.02 | 0.07 | 0.02 | 0.77 |
| 102 Dalmatians | 2000 | Oddball | Dalmatian | 2.14 | 0.02 | 0.01 | 0.90 |
| The Return of Rin Tin Tin | 1947 | Rin Tin Tin | German Shepherd | -2.26 | 0.11 | 0.06 | 0.93 |
| The Shaggy Dog | 1959 | Chiffonn | Old English Sheepdog | 2.09 | 0.07 | 0.05 | 0.94 |
| The Incredible Journey | 1963 | Bodger | Bull Terrier | 3.44 | 0.04 | 0.01 | 0.69 |

Although there was more than 1 case outside of the +/- 2.5 standardised score, the character’s cook’s distance was well below 1 and so was deemed to be having undue influence on the model. Bodger from *The Incredible Journey* (1963) was the only character that had a standardised residual greater than 3 which raises cause for concern. After investigating further, the breed registration data seems to be correct and responding to the film, but the character’s portrayal did not fit into the four hypothesises identified. Therefore, it may be that viewers are responding to the character in ways that was not captured in this study. There is no reason to believe the data point is an error and it is not placing undue influence on the model.

| Table 5 |  |  |  |
| --- | --- | --- | --- |
| *Checks carried out on Characters flagged as possible outliers* | |  |  |
| Tests | Bounds of acceptable values | Actual number of cases | Assumption Met |
| 95% of standardised residuals scores outside of -2 and +2 | 4.75 | 5 | Yes |
| 99% of standardised residuals scores outside of -2.5 and +2.5 | 0.95 | 2 | No |
| Investigate any standardised residual scores outside 3 | 0 | 1 | No |
| x2 Average Leverage | <0.06 | 0 | Yes |
| x3 Average Leverage | <0.09 | 0 | Yes |
| Covariance Ratio Lower Boundary | -0.91 | 0 | Yes |
| Covariance Ratio Upper Boundary | 1.09 | 0 | Yes |
| Cook's Distance | <1 | 0 | Yes |
| *Note.* Bounds of acceptable values and decision whether assumption has been met from Field, Miles & Field (2012) | | | |

Two Year Changes

| Table 6 |  |  |  |  |  |  |  |
| --- | --- | --- | --- | --- | --- | --- | --- |
| *Characters whose Standardised Residuals were less than -2 or greater than +2* | | | | | | | |
| Movie | Year | Character | Breed | Standardised Residuals | Cooks Distance | Leverage | Covariance Ratios |
| Trailing the Killer | 1932 | Lobo | German Shepherd | 2.59 | 0.21 | 0.08 | 0.90 |
| The Painted Hills | 1951 | Shep | Collie | -2.34 | 0.14 | 0.07 | 0.92 |
| The Lightning Warrior | 1931 | Rin Tin Tin | German Shepherd | 3.04 | 0.29 | 0.08 | 0.82 |
| 102 Dalmatians | 2000 | Dipstick | Dalmatian | 3.93 | 0.12 | 0.02 | 0.61 |
| 102 Dalmatians | 2000 | Oddball | Dalmatian | 4.11 | 0.08 | 0.01 | 0.57 |
| One Hundred and One Dalmatians | 1969 | Colonel | Old English Sheepdog | 2.62 | 0.09 | 0.04 | 0.85 |

Two characters from *102 Dalmatians* (2000) had large outliers but according to Cook’s Distance (see Table 6) is not placing undue influence on the model. The breed registrations of Dalmatians may have been affected outside what is expected because of the rerelease of the original *One Hundred and One Dalmatians* (1961) in 1991 and the release of the remake in 1996. Sheen (2005) notes that between 1996 and 2000, the Dalmatian image was used to sell ‘almost everything’. The combined effect of film and advertising is likely to create larger changes in the breed registration than what the model predicts. Therefore, this data point is correct and legitimate and should be retained.

| Table 7 |  |  |  |
| --- | --- | --- | --- |
| *Checks carried out on Characters flagged as possible outliers* | | | |
| Tests | Bounds of acceptable values | Actual number of cases | Assumption Met |
| 95% of standardised residuals scores outside of -2 and +2 | 4.75 | 6 | Yes |
| 99% of standardised residuals scores outside of -2.5 and +2.5 | 0.95 | 4 | No |
| Investigate any standardised residual scores outside 3 | 0 | 3 | No |
| x2 Average Leverage | <0.06 | 3 | No |
| x3 Average Leverage | <0.09 | 0 | Yes |
| Covariance Ratio Lower Boundary | -0.91 | 0 | Yes |
| Covariance Ratio Upper Boundary | 1.09 | 0 | Yes |
| Cook's Distance | <1 | 0 | Yes |
| *Note.* Bounds of acceptable values and decision whether assumption has been met from Field, Miles & Field (2012) | | | |

Five Year Changes

| Table 8 |  |  |  |  |  |  |  |
| --- | --- | --- | --- | --- | --- | --- | --- |
| *Characters whose Standardised Residuals were less than -2 or greater than +2* | | | | | | |  |
| Movie | Year | Character | Breed | Standardised Residuals | Cooks Distance | Leverage | Covariance Ratios |
| One Hundred and One Dalmatians | 1961 | Colonel | Old English Sheepdog | 2.95 | 0.13 | 0.04 | 0.79 |
| The Painted Hills | 1951 | Shep | Collie | -2.53 | 0.16 | 0.07 | 0.89 |
| 102 Dalmatians | 2000 | Dipstick | Dalmatian | 3.17 | 0.08 | 0.02 | 0.73 |
| 102 Dalmatians | 2000 | Oddball | Dalmatian | 3.33 | 0.06 | 0.02 | 0.70 |
| One Hundred and One Dalmatians | 1969 | Colonel | Old English Sheepdog | 2.05 | 0.06 | 0.04 | 0.93 |

Again, the characters from the 101 Dalmatians franchise created large residuals. As explained previously, the Dalmatian image was used repeatedly during the 1990s and 2000s and so this is likely adding to the number of people buying Dalmatians (Sheen, 2005). Old English Sheepdogs have also been used extensively in advertising which may be contributing to the high standardised residual. Old English Sheepdogs have been used in the paint maker Dulux’s advertisements since 1961 causing the breed to be nicknamed ‘Dulux Dog’ (Dulux, 2020). There is no reason to be believe that these data points were errors with Cook’s Distance well below 1 indicates that these are not placing undue influence on the model and should be retained.

| Table 9 |  |  |  |
| --- | --- | --- | --- |
| *Checks carried out on Characters flagged as possible outliers* | | |  |
| Tests | Bounds of acceptable values | Actual number of cases | Assumption Sufficiently Met |
| 95% of standardised residuals scores outside of -2 and +2 | 4.50 | 5 | Yes |
| 99% of standardised residuals scores outside of -2.5 and +2.5 | 0.90 | 3 | No |
| Investigate any standardised residual scores outside 3 | 0.00 | 2 | No |
| x2 Average Leverage | 0.07 | 0 | Yes |
| x3 Average Leverage | 0.10 | 0 | Yes |
| Covariance Ratio Lower Boundary | -0.90 | 0 | Yes |
| Covariance Ratio Upper Boundary | 1.10 | 0 | Yes |
| Cook's Distance | 1.00 | 0 | Yes |
| *Note.* Bounds of acceptable values and decision whether assumption has been met from Field, Miles & Field (2012) | | | |

Ten Year Changes

| Table 10 |  |  |  |  |  |  |  |
| --- | --- | --- | --- | --- | --- | --- | --- |
| *Characters whose Standardised Residuals were less than -2 or greater than +2* | | | | | | | |
| Movie | Year | Character | Breed | Standardised Residuals | Cooks Distance | Leverage | Covariance Ratios |
| One Hundred and One Dalmatians | 1961 | Colonel | Old English Sheepdog | 3.59 | 0.22 | 0.08 | 0.41 |
| The Painted Hills | 1951 | Shep | Collie | -2.22 | 0.13 | 0.12 | 0.84 |
| The Shaggy Dog | 1959 | Chiffonn | Old English Sheepdog | 2.77 | 0.13 | 0.08 | 0.65 |
| Fangs of the Wild | 1939 | Queeny | German Shepherd | 2.01 | 0.04 | 0.05 | 0.84 |

Again, the Old English Sheepdog appears as an outlier as it was used extensively in advertisements and so likely adding to the effect of the movies. Queeny from *Fangs of the Wild* (1939) was the love interest of Rin Tin Tin and so any rises in breed registrations relative to the overall dog registrations is likely to be also due to his portrayal in the movie as well as the breed’s popularity during the 1930’s. The Cooks Distance is extremely low and so not placing undue influence on the model. Therefore, all data points should be retained.

| Table 11 |  |  |  |
| --- | --- | --- | --- |
| *Checks carried out on Characters flagged as possible outliers* | | |  |
| Tests | Bounds of acceptable values | Number of cases fail test | Assumption Sufficiently Met |
| 95% of standardised residuals scores outside of -2 and +2 | 3.70 | 4 | Yes |
| 99% of standardised residuals scores outside of -2.5 and +2.5 | 0.74 | 2 | No |
| Investigate any standardised residual scores outside 3 | 0.00 | 1 | No |
| x2 Average Leverage | 0.14 | 0 | Yes |
| x3 Average Leverage | 0.20 | 0 | Yes |
| Covariance Ratio Lower Boundary | -0.80 | 0 | Yes |
| Covariance Ratio Upper Boundary | 1.20 | 0 | Yes |
| Cook's Distance | 1.00 | 0 | Yes |
| *Note.* Bounds of acceptable values and decision whether assumption has been met from Field, Miles & Field (2012) | | | |

***Variable Types***

All predictor variables are quantitative. The scores are recorded as proportions which are acceptable and commonly used when performing linear regression (Warton & Hui, 2011). Scores are inputted as percentages (e.g. Toto’s heroism score is entered as 17 and not 0.17) to aid with interpretation. All outcome variables are also quantitative and unbounded (Field et al., 2012).

***Non-Zero Variance***

The level of variation in predictors was checked and none have variances of zero and so this assumption has been met. (Field et al., 2012). The Nature/Society Boundary predictor is close to zero because few movies depicted dogs portraying this idea and so many characters scored zero. There were some dogs that did meet some of these criteria and so this predictor is retained in the analysis.

| Table 12 |  |  |  |
| --- | --- | --- | --- |
| *Amount of variance in each predictor* | | | |
| Dog Hero | Anthropomorphism | Western Ideals | Nature/Society Boundary |
| 0.05 | 0.07 | 0.06 | 0.02 |

***No*** ***Multicollinearity***

There is no perfect relationship between predictor variables, but they correlate significantly with each other as found in the correlation matrix.

| Table 13 |  |  |  |  |
| --- | --- | --- | --- | --- |
| *Correlation Matrix showing correlation between predictors* | | | | |
|  | Dog Hero | Anthro | Western Ideals | Nature/Society Boundary |
| Dog Hero | 1 | 0.38*** | 0.33 | 0.15 |
| Anthropomorphism | 0.38*** | 1 | 0.44 | -0.03 |
| Western Ideals | 0.33*** | 0.44*** | 1 | 0 |
| Nature/Society Boundary | 0.15 | -0.03 | 0 | 1 |

*Note.* * indicates *p* < .05. ** indicates *p* < .01. *** indicates *p* < .001

The Variation Inflation Factor (VIF) was calculated and used to determine if multicollinearity was present. As the VIF for all predictors was less than 10 for all periods, it suggests there is no multicollinearity present (see Table 14 for results) (Field et al., 2012). The tolerances for each change period was greater than 0.2, again providing evidence that multicollinearity is not an issue with the data (Field et al., 2012). Finally, the average VIF should be close to 1 to ensure the models will not be biased. The average VIF was 1.17 for 1, 2 and 5 year changes and 1.27 for 10 year changes suggesting the model is not overly biased (Field et al., 2012)

| Table 14 |  |  |  |  |  |  |
| --- | --- | --- | --- | --- | --- | --- |
| *Tests conducted to identify multicollinearity between predictors* | | | | | | |
| Test | Time Period | Ideal | Dog Hero | Anthro-  pomorphism | Western Ideals | Nature/Society Boundary |
| VIF | 1 | <10 | 1.17 | 1.17 | - | - |
|  | 2 |  | 1.17 | 1.17 | - | - |
|  | 5 |  | 1.17 | 1.17 | - | - |
|  | 10 |  | 1.28 | 1.39 | 1.34 | 1.06 |
| Tolerance | 1 | >0.2 | 0.86 | 0.86 | - | - |
|  | 2 |  | 0.86 | 0.86 | - | - |
|  | 5 |  | 0.85 | 0.85 | - | - |
|  | 10 |  | 0.78 | 0.72 | 0.75 | 0.94 |

***Homogeneity of Variance***

By visually inspecting Figure 4, the residuals at each level of the predictors for each period have the same variance across fitted values and so the assumption of homogeneity of variance has been met (Field et al., 2012).


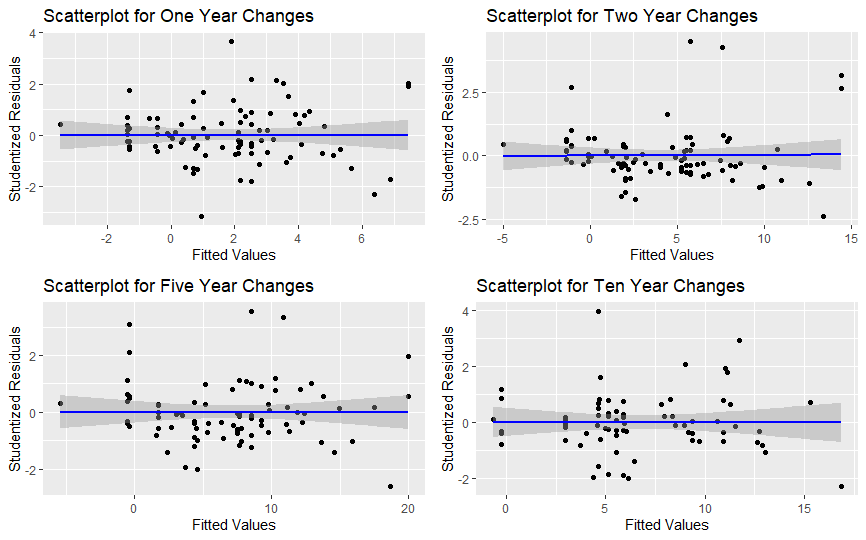


***Assumption of Independence of Error***

The models’ errors of One, Two- and Five-Year Changes were significantly correlated (Durbin-Watson test – One Year: *DW* = 1.6, *p* = 0.029, Two Year: *DW* = 1.6, *p* = 0.020, Five Year: *DW* = 1.66, *p* = 0.040) suggesting autocorrelation exists (Field et al., 2012). The data is likely a time series as a breed’s registrations are likely to be affected by previous years’ popularity.

To identify any significant autocorrelation, ACF graphs of each change period was visually inspected to provide an indication significant instances of autocorrelation. One- and Ten-Year Changes had no significant instances (the black lines did not cross the dotted blue lines) but Two and Five Year Changes did. Hyndman and Athanasopoulos (2018) warn however, that because each line is being tested independently, type 2 errors are common. This means that the graphs below are likely to show more significant results than are true. Therefore, portmanteau tests (tests that look at group autocorrelation rather than individual instances) need to be conducted to confirm if significant autocorrelation is present in the data (Hyndman & Athanasopoulos, 2018).


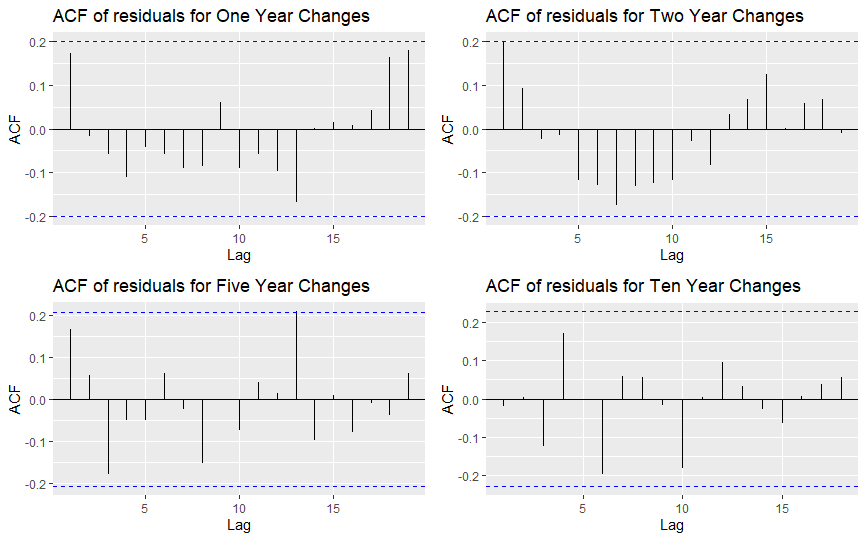


Tests were conducted to identify if the residuals could be distinguished from a white noise series (Hyndman & Athanasopoulos, 2018). If significant, it would indicate that the model is not a sufficient fit to the data because of a large amount of autocorrelation. The Box-Pierce, Box-Ljung and Breusch- tests were conducted, and all returned non-significant results indicating the model is a good fit for the data. See Table 15 for full results. Based on these tests, the assumption of independence of errors has been sufficiently met.

| Table 15 |  |  |  |  |  |
| --- | --- | --- | --- | --- | --- |
| *Tests conducted to identify significant levels of autocorrelation* | | | | | |
| Test | Time Period | Test Statistic | | Degrees of Freedom | p-value |
| Box-Pierce | 1 | X-Squared | 7.45 | 8 | 0.490 |
|  | 2 |  | 15.02 |  | 0.059 |
|  | 5 |  | 9.03 |  | 0.340 |
|  | 10 |  | 9.09 |  | 0.335 |
| Box-Ljung | 1 | X-Squared | 7.99 | 8 | 0.435 |
|  | 2 |  | 16.30 |  | 0.038 |
|  | 5 |  | 9.69 |  | 0.287 |
|  | 10 |  | 10.21 |  | 0.251 |
| Breusch-Godfrey^1^ | 1 | LM Test | 8.68 | 10 | 0.562 |
|  | 2 |  | 10.28 |  | 0.416 |
|  | 5 |  | 10.22 |  | 0.422 |
|  | 10 |  | 9.40 |  | 0.495 |
| *Note.* Tests and interpretation from (Hyndman & Athanasopoulos, 2018)  ^1^Breusch-Godfrey is a test specifically designed for regression | | | | | |

***Independence***

The independent variable was not completely independent from each other because some characters were nested within movies or breeds. However, there were only 2 movies that had characters who received the same scores, *The Doberman Gang* (1972) and *Oliver & Company* (1988) and so independence is unlikely to violate the assumption. The most egregious examples of independence, rereleases, which were the same characters, breeds and movies released at different time points, were tested to ensure they were not affecting results. The models were run with rereleases included and excluded but the results were the same. Therefore, it is expected that independence will not have a large effect and affect results.

***Assumption of Linearity***

By visually inspecting the plots of the residuals for each predictor, there is no clear pattern. This indicates that the data is linear (Hyndman & Athanasopoulos, 2018).


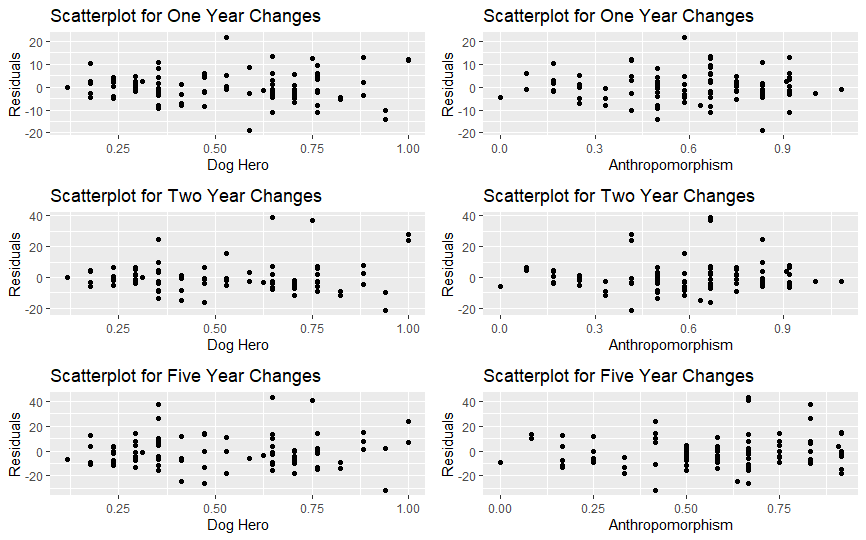


The ten-year changes do appear to have a pattern, especially Western Ideals, whose residuals are clustered around the low scores. Few movies in the sample had characters that were portrayed as a Nature/Society Boundary and so the majority of the scores were 0.

Ten-year changes


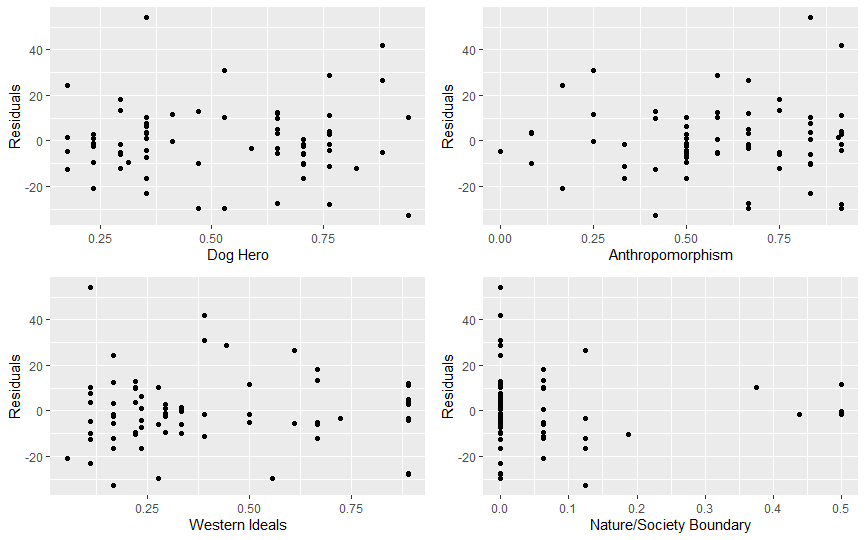


***Normally Distributed Errors***

Histogram of studentised errors are largely normally distributed for one, five- and ten-year change periods. However, there does appear to be some skew to the left of all graphs, especially for the two-year change period. Further tests were therefore required to determine the level of skew and if it is significant.


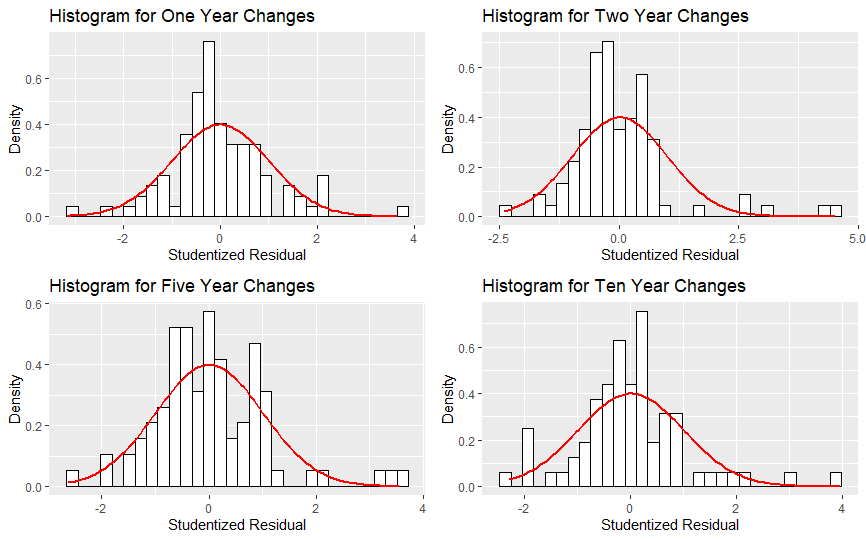


There is a significant amount of skew to the left for each time period (see Table 16) (Field et al., 2012). The Shapiro-Wilk tests confirms that the change periods are non-normal as the p-values are significant (*p* = 0.000). However, Field et al. (2012) explains that larger sample sizes can produce a significant result in the Shapiro-Wilk’s test when there is a small deviation from normal and so should always be used alongside histograms and Q-Q plots

| Table 16 |  |  |  |  |
| --- | --- | --- | --- | --- |
| *Tests to determine the normality of errors* | | |  |  |
| Test | 1 Year Changes | 2 Year Changes | 5 Year Changes | 10 Year Changes |
| skewness | 0.68 | 2.14 | 0.88 | 0.96 |
| skew.2SE | 1.38 | 4.33 | 1.77 | 1.94 |
| kurtosis | 1.44 | 6.09 | 1.14 | 2.15 |
| kurt.2SE | 1.47 | 6.21 | 1.16 | 2.19 |
| Shapiro-Wilk Test (*W*) | 0.94 | 0.78 | 0.94 | 0.92 |
| Shapiro-Wilk Test p-value | 0.000 | 0.000 | 0.000 | 0.000 |

The Q-Q Plots shows some deviations from normality at the top right of the line but is otherwise close to a straight line for each time period. Although caution should be taken when attempting to generalise the sample to the population of dog movies, the assumption of normality of errors has been sufficiently met to proceed with the multiple linear regression models.


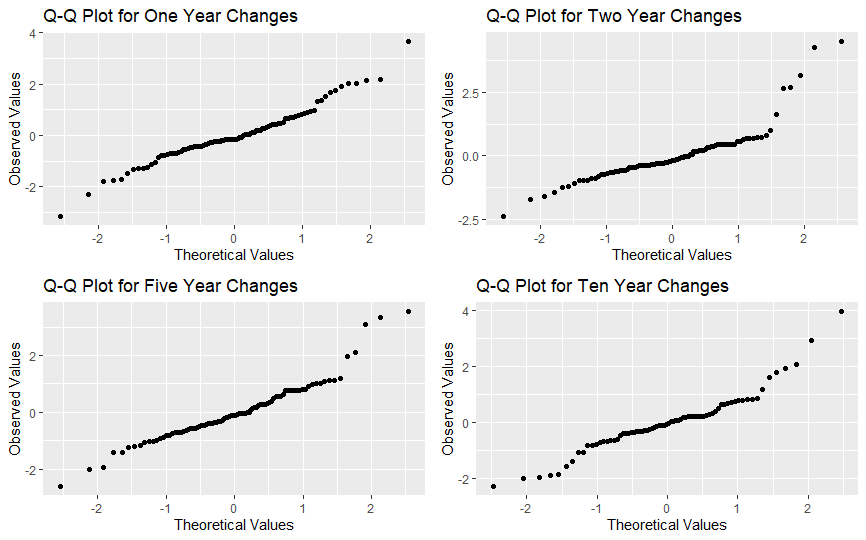


Code available at FigShare here

References

Dulux. (2020). It’s a (Dulux) dog’s life. Retrieved from <https://www.dulux.co.uk/en/articles/it%E2%80%99s-dulux-dog%E2%80%99s-life>

Field, A. P., Miles, J., & Field, Z. (2012). Discovering statistics using R/Andy Field, Jeremy Miles, Zoë Field. In: London; Thousand Oaks, Calif.: Sage.

Hyndman, R. J., & Athanasopoulos, G. (2018). *Forecasting: principles and practice*: OTexts.

Sheen, E. (2005). 101 and counting: Dalmatians in film and advertising. *Worldviews: Global Religions, Culture, and Ecology, 9*(2), 236-254.

Warton, D. I., & Hui, F. K. (2011). The arcsine is asinine: the analysis of proportions in ecology. *Ecology, 92*(1), 3-10.
