# Supplementary material for: The making of a (dog) movie star: The effect of the portrayal of dogs in movies on breed registrations in the United States
Source: PLoS One. 2022 Jan 12;17(1):e0261916. doi: 10.1371/journal.pone.0261916 (PMC8754329; doi:10.1371/journal.pone.0261916)
Supplement: S3 Appendix — (DOCX) [file pone.0261916.s007.docx]

## S3 Appendix. Results with rereleases excluded.

The steps for Multiple Linear Regression with rereleases excluded were followed as described in Field, Miles, and Field (2012). The results below have excluded the *One Hundred and One Dalmatians* (1961) and *Lady and the Tramp* (1955) rereleases.

| *ANOVA results to find the model that best fits the data* | | | | |  | |  | |  | |  |
| --- | --- | --- | --- | --- | --- | --- | --- | --- | --- | --- | --- |
| Time Period | Model Number | Residual Degrees of Freedom | Residual Sum of Squares | Degrees of Freedom | | Sum of Squares | | F Statistic | | p-value | |
| 1 Year Changes | Model 1 | 69 | 3432.2 |  | | | | | | | |
|  | **Model 2** | **68** | **3206.5** | **1.00** | | **225.69** | | **4.65** | | **0.035*** | |
|  | Model 3 | 67 | 3205.8 | 1.00 | | 0.70 | | 0.01 | | 0.905 | |
|  | Model 4 | 66 | 3204.7 | 1.00 | | 1.12 | | 0.02 | | 0.880 | |
| 2 Year Changes | Model 1 | 69 | 7845.6 |  | | | | | | | |
|  | **Model 2** | **68** | **7133.4** | **1.00** | | **712.19** | | **6.82** | | **0.011*** | |
|  | Model 3 | 67 | 6902.1 | 1.00 | | 231.33 | | 2.21 | | 0.142 | |
|  | Model 4 | 66 | 6896.5 | 1.00 | | 5.62 | | 0.05 | | 0.817 | |
| 5 Year Changes | Model 1 | 64 | 13713 |  | | | | | | | |
|  | **Model 2** | **63** | **12520** | **1.00** | | **1192.59** | | **5.92** | | **0.018*** | |
|  | Model 3 | 62 | 12327 | 1.00 | | 192.89 | | 0.96 | | 0.332 | |
|  | Model 4 | 61 | 12282 | 1.00 | | 45.62 | | 0.23 | | 0.636 | |
| 10 Year Changes | Model 1 | 48 | 13371 |  | | | | | | | |
|  | Model 2 | 47 | 13284 | 1.00 | | 87.12 | | 0.30 | | 0.589 | |
|  | Model 3 | 46 | 13281 | 1.00 | | 2.46 | | 0.01 | | 0.928 | |
|  | **Model 4** | **45** | **13235** | **1.00** | | **46.46** | | **0.16** | | **0.693** | |
| *Note. Bold indicates selected model and * indicates p < .05.* | | | | | | | | | | |  |

Like when rereleases were included, Model 2 was found to be the best fit for periods 1, 2 and 5 years, and so multiple regression was run for these. As no results were significant for 10-year change periods, Model 4 was run.

The portrayal of a dog represents 10.32% of the variance of AKC breed registrations for one year changes, *F*(2,68) = 3.91, *p* = .025. It represents 16.73% of the variance of AKC breed registrations for two year changes, *F*(2,68) = 6.83, *p* = .002, 14.99% for five year changes, *F*(2,63) = 5.56, *p* = .006. The portrayal of the dog did not have a significant effect on ten-year changes.

| *Multiple linear regression results when rereleases are excluded* | | | | |
| --- | --- | --- | --- | --- |
| Predictor | Estimate (b) | Std. Error | t-value | p-value |
| 1 Year Changes | | | | |
| Intercept | 0.00 | 2.23 | 0.24 | .814 |
| Dog Hero | 0.33 | 3.99 | 2.54 | .014* |
| Anthropomorphism | -0.28 | 3.31 | -2.19 | .032* |
| 2 Year Changes | | | | |
| Intercept | 0.00 | 3.33 | 0.10 | .919 |
| Dog Hero | 0.33 | 5.95 | 3.51 | .001*** |
| Anthropomorphism | -0.28 | 4.94 | -2.61 | .011* |
| 5 Year Changes | | | | |
| Intercept | 0.00 | 4.61 | 0.75 | .459 |
| Dog Hero | 0.42 | 8.35 | 3.15 | .002** |
| Anthropomorphism | -0.32 | 7.61 | -2.45 | .017* |
| 10 Year Changes | | | | |
| Intercept | 0.00 | 6.86 | 0.43 | .672 |
| Dog Hero | 0.24 | 12.60 | 1.44 | .158 |
| Anthropomorphism | -0.10 | 10.82 | -0.55 | .586 |
| Western Ideals | -0.01 | 14.14 | -0.06 | .950 |
| Nature/Society Boundary | -0.06 | 17.82 | -0.40 | .693 |
| *Note. * indicates p < .05., ** indicates p < .01, *** indicates p < .001* | | | | |

References

Field, A. P., Miles, J., & Field, Z. (2012). Discovering statistics using R/Andy Field, Jeremy Miles, Zoë Field. In: London; Thousand Oaks, Calif.: Sage.
