# Supplementary material for: The making of a (dog) movie star: The effect of the portrayal of dogs in movies on breed registrations in the United States
Source: PLoS One. 2022 Jan 12;17(1):e0261916. doi: 10.1371/journal.pone.0261916 (PMC8754329; doi:10.1371/journal.pone.0261916)
Supplement: S4 Appendix — (DOCX) [file pone.0261916.s008.docx]

# S4 Appendix. Movies Cited.

Movies cited below include those referred to in the text and those analysed for the study. Ordered alphabetically by the movie title.

Herek, S. (Director). (1996). *101 Dalmatians* [Motion picture]. United States: Buena Vista Pictures Distribution.

Lima, K. (Director). (2000). *102 Dalmatians* [Motion picture]. United States: Buena Vista Pictures.

Dyke, W. V. (Director), & Goodrich, F., Hackett, A., & Hammett, D. (Writers). (1936). *After the thin man* [Motion picture]. United States: Metro-Goldwyn-Mayer.

Smith, C. M. (Director), & Weinstein, H., & Weinstein, B. (Producers). (1997). *Air Bud* [Motion picture]. United States: Walt Disney Productions.

Levant, B. (Director). (1992). *Beethoven* [Motion picture]. United States: Universal Pictures.

Camp, J. (Director), & Camp, J., & Vanston, E. (Producers). (1974). *Benji* [Motion picture]. United States: Mulberry Square Productions.

Camp, B. (Director), & Blum, J. (Producer). (2018). *Benji* [Motion picture]. United States: Blumhouse Productions.

Guest, C. (Director), & Mark, G. (Producer). (2000). *Best in Show* [Motion picture]. United States: Castle Rock Entertainment.

Tokar, N. (Director), & Disney, W. (Producer). (1962). *Big Red* [Motion picture]. United States: Buena Vista Pictures Distribution.

Guterman, L. (Director). (2001). *Cats & Dogs* [Motion picture]. United States: Warner Bros. Pictures.

Teague, L. (Director), & Blatt, D. H. (Producer). (1985). *Cujo* [Motion picture]. United States: Sunn Classic Pictures.

Claxton, W. (Director), & Webb, H. S. (Producer). (1939). *Fangs of the Wild* [Motion picture]. United States: Metropolitan Pictures.

Chaffey, D. (Director), & Disney, W. (Producer). (1961). *Greyfriars Bobby: The True Story of a Dog* [Motion picture]. United States: Buena Vista Pictures Distribution.

Ellis, D. R. (Director), & Jossen, B. (Producer). (1996). *Homeward bound II, Lost in San Francisco* [Motion picture]. United States: Walt Disney Productions.

Dunham, D. (Director), & Disney, W. (Producer). (1993). *Homeward bound: The incredible journey* [Motion picture]. United States: Buena Vista Pictures Distribution.

Haid, C. (Director). (1994). *Iron Will* [Motion picture]. United States: Walt Disney Productions.

Hoffman, H. (Director), & Berman, H. (Producer). (1955). *It's a Dog's Life* [Motion picture]. United States: Metro-Goldwyn-Mayer.

King, H. (Director), Zanuck, D. F., & Johnson, N. (Producers), & Johnson, N. (Writer). (1939). *Jesse James* [Motion picture]. United States: Twentieth Century Fox.

Daniel, R. (Director), & Gordon, C., & Gordon, L. (Producers). (1989). *K-9* [Motion picture]. United States: Universal Pictures.

Disney, W. (Producer), & Luske, H., Geronimi, C., & Jackson, W. (Directors). (1955). *Lady and the Tramp* [Motion picture]. United States: Buena Vista Pictures Distribution.

Bean, C. (Director), & Sabatini, D. L. (Producer). (2019). *Lady and the Tramp* [Motion picture]. United States: The Walt Disney Company.

Wilcox, F. M. (Director), & Marx, S. (Producer). (1943). *Lassie come home* [Motion picture]. United States: Metro-Goldwyn-Mayer.

Kline, B. (Director), & Schary, D. (Writer). (1931). *Lightning Warrior, Ch 1: The Drums of Doom* [Motion picture]. United States: Mascot Pictures.

Landers, L. (Director), & MacDonald, W. (Producer). (1948). *My Dog Rusty* [Motion picture]. United States: Columbia Pictures.

Sanders, C. (Director), & Mangold, J., & Stoff, E. (Producers). (2020). The Call of the Wild [Motion picture]. United States: 20th Century Studios.

Scribner, G. (Director), & Disney, W. (Producer). (1988). *Oliver & Company* [Motion picture]. United States: Buena Vista Pictures Distribution.

Geronimi, C. (Writer), & Reitherman, W., & Luske, H. (Directors). (1961). *One hundred and one dalmatians* [Motion picture]. United States: Buena Vista Pictures Distribution.

Cohn, R. (Producer), & Jason, W. (Director). (1948). *Rusty leads the way* [Motion picture]. United States: Columbia Pictures.

Gosnell, R. (Director), & Barbera, J., Engelman, R., Hanna, W., Mason, A., & Smith-Wait, K. (Producers). (2002). *Scooby-Doo* [Motion picture]. United States: Warner Bros. Pictures.

Levant, B. (Director), & Disney, W. (Producer). (2002). *Snow Dogs* [Motion picture]. United States: Buena Vista Pictures Distribution.

Nigh, W. (Director), & Dunlap, S. R. (Producer). (1940). *Son of the Navy* [Motion picture]. United States: Monogram Pictures.

McEveety, V. (Director), & Disney, W. (Producer). (1972). *The Biscuit Eater* [Motion picture]. United States: Buena Vista Pictures Distribution.

Chudnow, B. (Director), & Chudnow, D., & Temaner, I. (Producers). (1972). *The Doberman Gang* [Motion picture]. United States: Rosamund Productions, Inc.

Markle, F. (Director), & Disney, W. (Producer). (1963). *The Incredible Journey* [Motion picture]. United States: Buena Vista Pictures Distribution.

Kress, H. F. (Director). (1951). *The Painted Hills starring Lassie* [Motion picture]. United States: Metro-Goldwyn-Mayer.

Rosen, M. (Director), & Eberts, J. (Producer). (1982). *The Plague Dogs* [Motion picture]. United States: Nepenthe Productions.

Nosseck, M., & Stephens, W. (Directors). (1947). *The Return of Rin Tin Tin* [Motion picture]. United States: Romay Pictures Inc.

Stevenson, R. (Director), & Tait, D. (Writer). (1976). *The Shaggy D.A.* [Motion picture]. United States: Buena Vista Distribution Company, Inc.

Barton, C. (Director), Disney, W., & Walsh, B. (Producers), & Walsh, B., & Hayward, L. (Writers). (1959). *The shaggy dog* [Motion picture]. United States: Buena Vista Distribution Co.

Dyke, W. V. (Director), & Stromberg, H. (Producer). (1931). *The Thin Man* [Motion picture]. United States: Metro-Goldwyn-Mayer.

Tokar, N., & Disney, W. (Directors). (1966). *The Ugly Dachshund* [Motion picture]. United States: Buena Vista Pictures Distribution.

Fleming, V. (Director), Langley, N., Ryerson, F., Woolf, E. A., Stothart, H., Harburg, E. Y., Arlen, H., . . . Sewell, B. (Writers), & LeRoy, M., Gibbons, C., Horning, W. A., Willis, E. B., Adrian, & Dawn, J. (Producers). (1939). *The wizard of Oz* [Motion picture]. United States: Metro Goldwyn Mayer.

Zeidman, B. F., & Hunt, C. J. (Producers), Raymaker, H. (Director), & Richards, J., Smith, W. C., Goodfriend, P., Meyer, A., Potoker, O., & Wineland, S. K. (Writers). (1932). *Trailing the killer* [Motion picture]. United States: B.F. Zeidman Productions Ltd.

Luby, R. S. (Director), & Weeks, G. W. (Producer). (1942). *War Dogs* [Motion picture]. United States: Monogram Pictures.

Winner, M. (Director). (1976). *Won Ton Ton: The Dog Who Saved Hollywood* [Motion picture]. United States: Paramount Pictures.
