# Supplementary material for: The making of a (dog) movie star: The effect of the portrayal of dogs in movies on breed registrations in the United States
Source: PLoS One. 2022 Jan 12;17(1):e0261916. doi: 10.1371/journal.pone.0261916 (PMC8754329; doi:10.1371/journal.pone.0261916)
Supplement: S6 Appendix — (DOCX) [file pone.0261916.s010.docx]

**S6 Appendix. Best Subsets and AIC Results.**

**AIC Results for Stepwise Model Selection**

The *Akaike* information criterion (AIC) and ANOVA both indicate that Model 2 (which includes Dog Hero and Anthropomorphism) is the best fit for the data for 1, 2 and 5 year changes. There are no significant results for 10 year changes.

Notes on reading tables below: The model with the lowest AICc is selected and bolded. K is the number of model parameters plus the intercept and Log Likelihood is how likely the model is based on the data used.

*1 Year Changes with Rereleases Included*

| Model number | K | AICc | AICc Delta | AIC Weight | Cumulative Weight | Log Likelihood |
| --- | --- | --- | --- | --- | --- | --- |
| Model 1 | 3 | 707.78 | 3.02 | 0.11 | 0.9 | -350.76 |
| **Model 2** | **4** | **704.76** | **0** | **0.5** | **0.5** | **-348.16** |
| Model 3 | 5 | 705.79 | 1.03 | 0.3 | 0.79 | -347.56 |
| Model 4 | 6 | 708.05 | 3.29 | 0.1 | 1 | -347.55 |

*2 Year Changes with Rereleases Included*

| Model number | K | AICc | AICc Delta | AIC Weight | Cumulative Weight | Log Likelihood |
| --- | --- | --- | --- | --- | --- | --- |
| Model 1 | 3 | 707.78 | 3.02 | 0.11 | 0.9 | -350.76 |
| **Model 2** | **4** | **704.76** | **0** | **0.5** | **0.5** | **-348.16** |
| Model 3 | 5 | 705.79 | 1.03 | 0.3 | 0.79 | -347.56 |
| Model 4 | 6 | 708.05 | 3.29 | 0.1 | 1 | -347.55 |

*5 Year Changes with Rereleases Included*

| Model number | K | AICc | AICc Delta | AIC Weight | Cumulative Weight | Log Likelihood |
| --- | --- | --- | --- | --- | --- | --- |
| Model 1 | 3 | 727.92 | 3.53 | 0.1 | 0.93 | -360.82 |
| **Model 2** | **4** | **724.38** | **0** | **0.61** | **0.61** | **-357.96** |
| Model 3 | 5 | 726.46 | 2.08 | 0.22 | 0.82 | -357.88 |
| Model 4 | 6 | 728.64 | 4.26 | 0.07 | 1 | -357.82 |

*10 Year Changes with Rereleases Included*

| Model number | K | AICc | AICc Delta | AIC Weight | Cumulative Weight | Log Likelihood |
| --- | --- | --- | --- | --- | --- | --- |
| **Model 1** | **3** | **621.12** | **0** | **0.52** | **0.52** | **-307.39** |
| Model 2 | 4 | 622.23 | 1.11 | 0.3 | 0.81 | -306.82 |
| Model 3 | 5 | 623.69 | 2.57 | 0.14 | 0.96 | -306.4 |
| Model 4 | 6 | 626.03 | 4.91 | 0.04 | 1 | -306.39 |

Original table for comparison.

| Table 3 |  |  |  |  |  |  |  |
| --- | --- | --- | --- | --- | --- | --- | --- |
| *ANOVA results to find the model that best fits the data* | | | | | | | |
| Time Period | Model Number | Residual Degrees of Freedom | Residual Sum of Squares | Degrees of Freedom | Sum of Squares | F Statistic | p-value |
| 1 Year Changes | Model 1 | 93 | 3943.3 |  | | | |
|  | **Model 2** | **92** | **3744.4** | **1** | **198.88** | **4.79** | **0.031*** |
|  | Model 3 | 91 | 3738.9 | 1 | 5.51 | 0.13 | 0.717 |
|  | Model 4 | 90 | 3737.1 | 1 | 1.87 | 0.05 | 0.832 |
| 2 Year Changes | Model 1 | 93 | 8958.9 |  | | | |
|  | **Model 2** | **92** | **8481.6** | **1** | **477.34** | **5.13** | **0.026*** |
|  | Model 3 | 91 | 8375.4 | 1 | 106.22 | 1.14 | 0.288 |
|  | Model 4 | 90 | 8373 | 1 | 2.34 | 0.03 | 0.874 |
| 5 Year Changes | Model 1 | 88 | 15997 |  | | | |
|  | **Model 2** | **87** | **15011** | **1** | **985.97** | **5.60** | **0.020*** |
|  | Model 3 | 86 | 14984 | 1 | 27.23 | 0.15 | 0.695 |
|  | Model 4 | 85 | 14964 | 1 | 19.86 | 0.11 | 0.738 |
| 10 Year Changes | Model 1 | 72 | 17570 |  | | | |
|  | Model 2 | 71 | 17305 | 1 | 265.82 | 1.07 | 0.30 |
|  | Model 3 | 70 | 17109 | 1 | 196.00 | 0.79 | 0.38 |
|  | **Model 4** | **69** | **17101** | **1** | **7.16** | **0.03** | **0.87** |
| *Note. Bold indicates selected model and * indicates p < .05.* | | | | | | | |

**Best Subset Method**

We then ran a Best Subset method of model selection to ensure our a priori selection criteria did not bias results. Best Subsets runs all possible combinations of models for a certain number of predictors and selects the best model. It does this until it reaches the maximum number of predictors that could be entered. For example, it will run all possible models when 1 predictor is added to the model and returns the best, it then does the same for 2 predictors added, until 4 predictors are added.

To determine which was the best model for the data, we then used adjusted R^2^, Mallows’ CP and Schwartz's information criterion (BIC) (to be more conservative than an AIC) to compare models. We found that each of these methods found that model 2 was the best fit for the data (except for Adjusted R^2^ for 2-year changes). If there was disagreement between model selection tests, we referred to the most conservative method.

By rerunning our analysis using the Best Subset method of model selection, we found that the results matched those of our original Stepwise method. This confirms that our a priori selection criteria did not introduce bias.

***One Year Changes***

| The best models for each number of predictors entered | | | | | |
| --- | --- | --- | --- | --- | --- |
|  |  | Dog Hero | Anthropomorphism | Western Ideals | Nature/Society Boundary |
| 1 | (1) | "*" | " " | " " | " " |
| 2 | (1) | "*" | "*" | " " | " " |
| 3 | (1) | "*" | "*" | "*" | " " |
| 4 | (1) | "*" | "*" | "*" | "*" |

Note: Asterisk indicates that this variable was included in the model. A maximum of 4 predictors were entered resulting in 4 of the best models for each model size.

*Model comparison results*

To determine which model is the best fit for the data, Adjusted R^2^, Mallows’ CP and BIC were used. These tests all indicted that Model 2 was the best model for the data. This confirms our original analysis.

| Adjusted R^2^ | Mallows' Cp | BIC |
| --- | --- | --- |
| Model 2 | Model 2 | Model 2 |

***Two Year Changes***

| The best models for each number of predictors entered | | | | | |
| --- | --- | --- | --- | --- | --- |
|  |  | Dog Hero | Anthropomorphism | Western Ideals | Nature/Society Boundary |
| 1 | (1) | "*" | " " | " " | " " |
| 2 | (1) | "*" | "*" | " " | " " |
| 3 | (1) | "*" | "*" | "*" | " " |
| 4 | (1) | "*" | "*" | "*" | "*" |

Note: Asterisk indicates that this variable was included in the model. A maximum of 4 predictors were entered resulting in 4 of the best models for each model size.

*Model comparison results*

| Adjusted R^2^ | Mallows' Cp | BIC |
| --- | --- | --- |
| Model 3 | Model 2 | Model 2 |

As 2 of the 3 methods indicate Model 2 is the best model for the data, and these are more conservative by penalising the model harsher for additional predictors, Model 2 is the best model for the data.

***Five Year Changes***

| The best models for each number of predictors entered | | | | | |
| --- | --- | --- | --- | --- | --- |
|  |  | Dog Hero | Anthropomorphism | Western Ideals | Nature/Society Boundary |
| 1 | (1) | "*" | " " | " " | " " |
| 2 | (1) | "*" | "*" | " " | " " |
| 3 | (1) | "*" | "*" | "*" | " " |
| 4 | (1) | "*" | "*" | "*" | "*" |

Note: Asterisk indicates that this variable was included in the model. A maximum of 4 predictors were entered resulting in 4 of the best models for each model size.

*Model comparison results*

| Adjusted R^2^ | Mallows' Cp | BIC |
| --- | --- | --- |
| Model 2 | Model 2 | Model 2 |

These results match with our original Stepwise and ANOVA analysis.

***Ten Year Changes***

| The best models for each number of predictors entered | | | | | |
| --- | --- | --- | --- | --- | --- |
|  |  | Dog Hero | Anthropomorphism | Western Ideals | Nature/Society Boundary |
| 1 | (1) | "*" | " " | " " | " " |
| 2 | (1) | "*" | "*" | " " | " " |
| 3 | (1) | "*" | "*" | "*" | " " |
| 4 | (1) | "*" | "*" | "*" | "*" |

Note: Asterisk indicates that this variable was included in the model. A maximum of 4 predictors was entered resulting in 4 of the best models for each model size.

*Model comparison results*

| Adjusted R^2^ | Mallows' Cp | BIC |
| --- | --- | --- |
| Model 2 | Model 1 | Model 1 |

These results are comparable with our original ANOVA as 10-year changes results are not significant.
