# Supplementary material for: The making of a (dog) movie star: The effect of the portrayal of dogs in movies on breed registrations in the United States
Source: PLoS One. 2022 Jan 12;17(1):e0261916. doi: 10.1371/journal.pone.0261916 (PMC8754329; doi:10.1371/journal.pone.0261916)
Supplement: Attachment — Submitted filename: Weir_Kessler_2021_ResponseToReviewers.docx [file pone.0261916.s011.docx]

*Manuscript PONE-D-20-34744*

*Response to Reviewers*

*Dear PLOS ONE editorial team*

*We thank the reviewers for their constructive comments. We believe that we have addressed them all and that it has produced a clearer and improved paper.*

*Our comments are below in blue.*

*Sincerely,*

*Sarah Weir and Sharon Kessler*

**Reviewers Comments to the Author**

**Reviewer #1**

This is a well written article that examines the relationship between the depiction of dog breeds in films in the United States and their popularity following these productions. The abstract and introduction addresses and summarises key points with a thorough review of existing literature and the gap for the current study. The research methods are robust with established and measurable results being produced that supports the conclusions. The discussion remains relevant to the produced results and considers the implications these can have on animal welfare with an acceptable discussion of potential limitations of the study. The article is presented in an intelligible manner which guides the reader throughout. An extensive range of supporting literature has been utilised throughout the piece.

*Authors’ response: Thank you!*

Few minor revisions are required,

1. care with the use of first person in a research piece (65, 524).

*Authors’ response: The use of the first person has been removed from the paper.*

2. ‘Breed standards’ (152) are mentioned but not noted as to where they originated from and who establishes these - useful to clarify that these are created by the AKC.

*Authors’ response: We have added a sentence into the methods (page 10, line 217), stating that we are using the breed standards as defined by the AKC.*

3. For the research methodology, please define how the chosen films were ‘randomly selected’ (383), explain how this has been carried out equally with bias removed.

*Authors’ response: We have added a description of this into the methods on pages 11 (lines 223 -230). Specifically, we used stratified random sampling by decade. The random sampling was carried out by SW, who scrolled through a list with her eyes closed, stopping randomly. While we don’t expect that this inadvertently introduced any biases, in the future, we would use a random number generator to select movies from a list.*

4. Lastly, I would advise ensuring that there is clear distinction as to when you are speaking about UK statistics and literature vs. US in the paragraph ‘False expectations of dog behaviour’.

*Authors’ Response: We have gone through the paragraph and indicated which statistics and literature are from the US and which are from the UK.*

**Reviewer #2:** The paper concerns an important area of research: The effect of media-representations, in this case films, on people’s tendency to buy dogs of specific breeds and their expectations in relation to their future companion. However, the paper has a number of flaws and limitation. I could spend days describing these but will only focus at the four most important:

1. There is a lack of connect between two of the main issues developed at great length in the introduction and what is actually studied and presented in the paper. In the introduction there is long presentation of how dogs end up being relinquished and have health problems related to breeding. Therefore, one would expect that the study should focus on the link between the breeds promoted in films and whether the purchase of these specific breeds leads to higher levels of relinquishments (for example because these breeds are more difficult to keep as companions), or to more health problems in companion dogs (because these breeds are more prone to breeding related health problems). However, whether this is the case is not really focussed on in the results- and discussion sections.

*Authors’ response: We thank the reviewer for the comments on the inclusion of relinquishment and health problems that we included in the paper. We had intended this to provide context on how the portrayal of dogs in movies can have real world consequences for dogs. However, we recognise that this was distracting and have streamlined the introduction to make it clearer that we are focusing and studying how portrayals of dogs may influence breed registrations.*

2. Another issue raised in the introduction is dogs being reared under suboptimal conditions at so-called puppy mills. Here it seems plausible that a sudden popularity of a certain breed could lead to increased demand of dogs from puppy mills. However, there are other reasons why people buy dogs from puppy mills – not least having to do with the price of the dogs – which are not discussed. And there is no real discussion of how the demand of specific breeds due to media exposure has affected the supply of these dogs. This is so for two reasons: Firstly, the demand of dogs is measured by means of development of number purebred dogs with pedigrees registered in AKC – so the supply from puppy mills does not enter the picture. Secondly, the results presented are not really focussed on breeds but rather on four so-called key themes.

*Authors’ response: We agree with the reviewer that the discussion of puppy mills is not directly relevant to the paper, so we have removed it. We also clarify that we are using the key themes to link* ***how*** *a dog is portrayed in a movie to an increase in breed registrations for* ***the particular breed shown in the movie.*** *We do this because it allows us to link the portrayal with an increase in demand for that breed specifically – as opposed to any dog or dogs in general. We have clarified this on page 9, lines 175 – 177 in the section “A Portrayal’s Effect on Breed Registrations”.*

3. A major shortcoming of the presentation of findings relating to relinquishments, extreme breeding and puppy mills presented in the introduction and in the discussion is that these are not based on proper literature reviews but on very selective, often partisan sources. Sometimes obviously wrong and absurd claims are made, like in the following sentences: “… a third of dogs are abandoned or relinquished to shelters before their second birthday [21, 22]. …. The average number of years a dog in the USA spends in their homes is only two years [21].”

*Authors’ response: As part of streamlining and focusing the paper, we have removed the emphasis on relinquishments, extreme breeding, and puppy mills. Thus, the problematic sources are no longer present in the paper. Moreover, we have checked the remaining sources to ensure they are based on robust, peer-reviewed studies and when available, include conflicting points of view.*

4. The paper claims to make an advance compared to a previous study in the field made by Ghirlanda et al. (2009). However, the main finding does not seem to add much to what was previously found: “… that the media is an important driver in how prospective dog owners choose a breed and suggests there is a real effect occurring.” The only difference found may by the author’s own admission be due to a loss in power in the new study.

*Authors’ response: Our study builds on the findings of Ghirlanda et al. 2014 in two ways: 1) We analyze changes in breed registrations according to key themes in the movies. This is beneficial because our findings allow us to begin to understand that some portrayals (i.e., dog hero) may increase demand, while others (i.e., anthropomorphism) may decrease it. This nuanced understanding of how portrayals may influence demand clearly goes beyond the analysis that Ghirlanda et al did. 2) While we used similar methods as Ghirlanda et al. 2014 to measure changes in breed registrations, we modified the methods to compare the changes of a given dog breed over time* ***relative to the changes of all dog breeds over that same time period.*** *This also builds on Ghirlanda et al. because it allows us to distinguish between general changes in demand for pedigreed dogs and the breed in question.*

*We believe that Ghirlanda et al. was a very insightful study, therefore our study seeks to continue this line of research and build on it’s findings. We thank the reviewer for explaining that we were not clear enough about the study’s aims and methods. We have streamlined our introduction to make it clearer on how our study builds on Ghirlanda et al. (2014)’s original study and how our study is significantly different.*

1. Reviewer #3: This manuscript interested me as it has been an area which has puzzled me for a while and I have been involved in researching. I hoped that it would be an in depth, welcome addition to the literature. However I can see some major shortcomings in the manuscript which I found difficult to look past, and as such I am sorry that I cannot recommend it for publication in its current form. I have tried to add some points in below which may be useful for you.

   Your introduction is VERY long and would benefit hugely from being cut down- it reads almost like a narrative.

*Authors’ response: We have significantly cut down the introduction and completely agree that it was very long. We have cut it down from the original 42 pages to now only 6.*

2. It flits between the UK and the Us, and it is very difficult to digest.

*Authors’ response: We have gone through the manuscript and clarified which country we are talking about.*

3. It goes from ownership to disease, through puppy mills, genetics etc. This is almost a literature review in itself. Please can we focus it on what is needed to introduce this study- not the entire dog research. You could have a nice literature review on dogs in movies, but the endless examples here make it difficult to read. The wander off into white supremacism and Nazis is not really needed here and is not that relevant to the study as far as I can see. Much of the data in the introduction seems irrelevant, and talks a lot about relinquishment of dogs, and this never comes through in the results of the discussion. Which I was disappointed at.

*Authors’ response: We agree with the reviewer that we included too much tangential information and that it was distracting. We have removed the discussions of puppy mills, genetics, white supremacy, and relinquishment. We have also added a short discussion of possible links with dog relinquishment in the discussion, as requested by the reviewer (Discussion, subsection “Conclusion,” page 29-30).*

4. Your literature survey does not appear to conform to the PRISMA study, and the data which you have provided is not enough for someone to repeat this study.

*Authors’ response: We have rewritten this section of the methods, adding the missing details and demonstrating that repeating the literature survey using PRISMA methods produces papers which generate the same themes (page 10, line 202 and S1 File, Sheet ‘Final Papers’).*

*We have also added a detailed account of how the papers were retrieved (page 9, lines 184-188), screened (page 9, lines 189-193), and analysed (page 9, lines 193 - 199). In addition, we provide the raw data in supplementary file S1 which includes all the details of the original review and the more recent PRISMA review. We believe this makes for a much clearer, more reproducible study and thank the reviewer for the advice. If the reviewer still feels there is anything missing, please let us know and we will be happy to include it.*

5. It would almost be better to look at numbers of animals of a given breed after a film and how that increases their adoption

*Authors’ response: We have clarified that we are focusing on how the portrayal of dogs in film (i.e., the key themes) alters breed registrations of the dog shown in the movie. We are using breed registrations as a proxy for demand (page 9, lines 175-177).*

6. Puppy mills are very good at increasing the numbers of dogs, such as when there is a huge demand like after a film. But much of the reason is due to prices, which seem to be missed in this study.

*Authors’ response: We realise now that our focus on puppy mills is tangential to the study and distracting to readers, so we have removed it. Thus, this comment no longer applies.*

7. I am also surprised at the lack of mention of how breed demand alters with dogs in films

*Authors’ response: We have clarified that we are focusing on how the portrayal of dogs in film (i.e., the key themes) alters breed registrations of the dog shown in the movie. We are using breed registrations as a proxy for demand (page 9, lines 175-177).*

8. The discussion is very similar to the introduction, and seems very repetitive and over long.

*Authors’ response: We have reduced and revised the introduction and discussion. They are now shorter and not repetitive.*

9. I also really struggled to see how this paper built on that of Ghirlanda et al. It seems to almost be that the findings here are the same as those of the previous study?

*Authors’ response: Our study builds on the findings of Ghirlanda et al. 2014 in two ways: 1) We analyze changes in breed registrations according to key themes in the movies. This is beneficial because our findings allow us to begin to understand that some portrayals (i.e., dog hero) may increase demand, while others (i.e., anthropomorphism) may decrease it. This nuanced understanding of how portrayals may influence demand clearly goes beyond the analysis that Ghirlanda et al did. 2) While we used similar methods as Ghirlanda et al. 2014 to measure changes in breed registrations, we modified the methods to compare the changes of a given dog breed over time* ***relative to the changes of all dog breeds over that same time period.*** *This also builds on Ghirlanda et al. because it allows us to distinguish between general changes in demand for pedigreed dogs and the breed in question.*

*We believe that Ghirlanda et al. was a very insightful study, therefore our study seeks to continue this line of research and build on its findings. We thank the reviewer for explaining that we were not clear enough about the study’s aims and methods. We have streamlined our introduction to make it clearer on how our study builds on Ghirlanda et al. (2014)’s original study and how our study is significantly different.*

10. There are some very odd comments, which don’t seem to fit the study, such as the abandonment of a third of dogs before they are 2? That is a huge number which doesn’t really seem to fit the requirements for this paper

*Authors’ response: As part of reducing and streamlining the paper, we have removed these comments.*

11. I have some real concerns about this manuscript, and I think it will need a major rewrite and focussing for it to be publishable I am afraid to say

*Authors’ response: We have significantly rewritten and refocused the paper, including addressing all of the comments raised by the reviewers. We believe it now makes a clear contribution to our understanding of how the portrayal of dogs in movies may influence breed registrations and hope that the reviewers will find it to be publishable.*
