# Supplementary material for: The making of a (dog) movie star: The effect of the portrayal of dogs in movies on breed registrations in the United States
Source: PLoS One. 2022 Jan 12;17(1):e0261916. doi: 10.1371/journal.pone.0261916 (PMC8754329; doi:10.1371/journal.pone.0261916)
Supplement: Attachment — Submitted filename: Weir_Kessler_2021_ResponseToReviewers.docx [file pone.0261916.s012.docx]

*Manuscript PONE-D-20-34744*

*Response to Reviewers*

*Dear PLOS ONE editorial team*

*We thank the reviewers for their constructive comments. We believe that we have addressed them all and that it has produced a clearer and improved paper.*

*Our comments are below in blue.*

*Sincerely,*

*Sarah Weir and Sharon Kessler*

**Comments to the Author**

**Reviewer #1**

The manuscript explores an interesting question: while we know that the presence of dog breeds in movies influences their successive popularity, is there an effect of *how* the dogs are portrayed? The manuscript goes in the right way to address the question, but it has in my opinion some issues related to clarity, especially related to the analysis, that need to be addressed before it can be considered for publication. Below I outline the ones I noticed.

Lines 203-204: a transition is missing, in the writing, between how the categories were formed and the hypotheses tested.

*Authors response: Thank you for highlighting this. We have added in a transition on page 10, line 203.*

Hypotheses: it seems strange that, given the initial question, all the hypotheses predict a positive effect of the categories on breed registrations (when would we expect negative effects?)

*Authors response: Thank you, this is an interesting question. All our hypothesises predict a positive effect because during the literature review we found most, if not all, dogs discussed were depicted as likeable characters. Also, a high proportion of movies that met our inclusion criteria were classed as ‘Family’ or were for children. Therefore, we expected that the portrayals of dogs in movies would generally be positive and would elicit a positive response from moviegoers.*

*However, we think it would be interesting for future research to better understand what kind of portrayals would have a negative effect on breed registrations. When watching the films, we found that there were some dog breeds depicted as villains (for example the human villain’s Dobermans in Oliver and Company (1988)), however these dogs were not on screen long enough to be included in the present study (were present for less than 5 minutes of screentime). We did have one villain dog, Cujo, however his character was associated with an increase of breed registrations of St. Bernard’s relative to all registrations after the movie. Future studies could create more appropriate coding criteria for villain dogs or perhaps look at other animals kept as pets who are viewed less positively by the public (e.g. snakes).*

Lines 311-321: I would take out this paragraph and simply say that the assumptions were met, pointing the reader to the Appendix. (Also, some parts read strange, such as "The models’ errors were found to be significantly correlated, suggesting the data were time series." )

*Authors note: We have removed this paragraph and have included the following in the main body on page 15, line 312 – 313,*

*“The assumptions of a multiple linear regression model were met, and results of tests are included in Appendix S5.”*

Hypothesis Testing: It was very difficult for me to figure out the point of the discussion in this section. It needs to be clearly stated at the beginning that they are deciding which of the four categories is more important.

*Authors note: We have rewritten this section and clearly outlined that we are deciding the importance of the predictors and how we calculated this. See page 15, lines 313 - 322.*

*Following additional suggests from the reviewers (see below), we did run a best subsets analysis, which did not make a piori decisions about which predictors were important, and the results confirmed our initial analysis.*

Mostly, why did the authors not use a multilevel model? This would have make possible analysing the effects of all the predictors without choosing a priori the ones that are more important. This is an important point that needs to be addressed by the authors.

*Authors note: We ran multiple linear regression because our data is not multilevel with a hierarchical structure. At first appearance our data appears multilevel because dogs seem to be nested in movies. However, we found that dog characters within a movie are not more similar than dog characters between movies. We have graphed this below.*

*In addition, the presence of remakes or sequels may appear to have the same dog across multiple movies, however these characters are a minority in the data and scored differently in each movie. Of the 48 movies, 6 characters featured in more than 1 movie because of either remakes or sequels. Of these only Rusty in his movies, “Rusty Leads the Way” and “My Dog Rusty” has some of the same scores (Hero = 47% vs 65%, Anthropomorphism = 42% vs 42%, Western Ideals = 22% vs 22% and Nature / Society Boundary = 0% vs 6%). We do not believe that this character would significantly change the results and so we believed that multiple linear regression was the most appropriate method.*

*We wanted to avoid overfitting a model with data that did not have a clear multilevel structure and so we used a linear multiple regression.*

Lines 352-359: "Each model with an additional predictor was then tested using an ANOVA to determine if these additions significantly improved the model fit." Wouldn't be AIC more appropriate?

*Authors note: We chose an ANOVA because we wanted to know which model significantly improved model fit. To ensure using an ANOVA did not bias our results, we calculated the AIC for each of our 4 models and years. The model with the lowest AIC is to be selected and so we were able to compare if the AIC and ANOVA directed us to select the same model. We found that when using the original Hierarchical Stepwise Model Building Approach, AIC results indicated that model 2 which includes Dog Hero and Anthropomorphism was the best fit for the data.*

*Then, to make sure our a priori approach hadn’t missed a model we hadn’t tested, we then ran the Best Subsets method and used Adjusted R^2^, CP, BIC (because its more conservative than AIC) to assess which model is the best fit for the data. Again model 2 was found to be the most appropriate (except for 10 year trends where no results were significant). We have added the following to the manuscript on page 19, lines 384 – 389 so it is clear for the reader that the analysis choices did not bias results:*

*“**To confirm the Hierarchical Stepwise a priori approach using an ANOVA to compare models was not biasing the results, AICs were calculated for the models created using a Stepwise model. A Best-Subsets approach was also run to ensure the a prior predictor order was not biasing results, and the resulting models were compared using a adjusted R^2^, CP’s Mallow and BIC criterion. These approaches confirmed the original findings (See S10 Appendix for results).”*

Lines 367-368: I would exclude from the analysis mentions to "trends toward significance".

*Authors note: We have removed mentions to “trends toward significance” (see page 17, line 357, page 20, lines 401 - 405 and page 22, lines 452 - 454).*

Results: It makes more sense to present first the main results, and subsequently the results concerning the exploratory analyses, i.e. I would start from the section "Hypothesis Testing".

*Authors note: We agree and have moved the Characters Analysed section below Hypothesis Testing. We have left the distribution of scores above Hypothesis Testing because it is descriptive data that we believe helps the reader understand the results of the hypotheses.*

Lines 449-452: This seems a repetition of what was already said at lines 440-443.

*Authors note: We have removed the repetitive information and retained only the information required to understand the figure. The caption on page 22, lines 443 - 446 now reads as*

*“Female dogs analysed were significantly more likely to have a higher score for Western Ideals. There were 3 male characters who were outliers, all from the One Hundred and One Dalmatians franchise; Pongo from One Hundred and One Dalmatians (1961), Pongo from the 1991 remake and Dipstick from 102 Dalmatians (2000).”*

Lines 457-459: These lines are unclear.

*Authors note: Thank you for pointing this out. We have changed these lines to the following on page 22 line 448 - 450:*

*“Wilcox rank sum tests were used to test if dogs were portrayed differently depending on whether the movie was live action or animated. These tests were used because normality tests were significant, p < 0.05”*

**Reviewer #2**

This is a well written and interesting manuscript which investigates how dogs are portrayed in movies and the effect which this has on their popularity. I only have a few minor comments to make to the authors which is indicative of the quality of the manuscript.

*Authors note: Thank you!*

Line 24- I am interested in why you chose the American KC, rather than the UK KC with you based in the UK? Was there a specific reason?

*Authors note: We decided to choose the American KC for a few reasons:*

1. *Previous research was focused on the US and we wanted to extend this research. We believed using the same registration data would make the results more comparable to existing literature*
2. *The Kennel Club data was freely available on the AKC digital archive and American dog movies were accessible through streaming platforms*
3. *Hollywood and American films have historically had a substantial, global cultural impact and so we believed focusing on films that had global reach would create more impactful results. Some of the most popular and influential dog movies of the century were created in the US (Lassie, Rin Tin Tin, Rinty franchises and all of the Disney movies).*

*It would be interesting for future research to use a similar approach in other countries to understand if this is a global phenomenon or if dogs are portrayed differently in different cultures.*

You have a few capital letters dotted throughout it which are not needed, and also sometimes Western is capitalised and not others. Please try to be consistent. Do white and black need capitalising?

*Authors note: Thank you for highlighting this. We have capitalised the Western because it is referring to a geographic place and the ideals is now consistently lowercase. However, we have capitalised all names of the hypothesises to make it clear when we are referring to these. We have made this clearer when outlining the hypothesises (page 10, lines 204 - 215). We have gone through and checked the other hypothesises to ensure they meet this rule.*

*We capitalised Black and White to conform to the APA Style Guidelines which can be found* [*here*](https://apastyle.apa.org/style-grammar-guidelines/bias-free-language/racial-ethnic-minorities)*.*

Line 61- comma after date

*Authors note: Added, thank you. See page 4, line 61.*

Line 64- comma after methods)

*Authors note: Added, thank you. See page 4, line 64.*

Line 93- full stop needed at the end of the sentence

*Authors note: Added, thank you. See page 5, line 93.*

Line 102- maybe placed on an equal footing?

*Authors note: Added, thank you. See page 6, line 102.*

You have a few bits in bold and underlined, and italicised. Please remove if not needed

*Authors note: We have removed any stylised elements from the paper. The only remaining words that are italicised are movies names to conform to APA style rules for writing about movies* [*here*](https://apastyle.apa.org/style-grammar-guidelines/italics-quotations/italics)*.*

Line 168- please reword this as it seems a bit strange

*Authors note: Thank you, we have rewritten this section on page 9, lines 166 - 168 as:*

*“For example, in John Carpenter’s The Thing (1982), the seemingly friendly husky dog at the beginning of the movie is actually an alien that absorbs everything in its path.”*

Line 293- comma after paper

*Authors note: Added, thank you. See page 14, line 294.*

Can you please use 0.005 (as an example for the p value. I.e. have a 0 before the decimal point

*Authors note: We have added a zero before every decimal point throughout the manuscript.*

Line 393- out of interest, do you know the sex of the dog? Could male dogs play a female role and vice versa?

*Authors note: Yes, we often know the sex of the character and can usually find out the sex of the dog actor playing the role. Sometimes the sex of the dog actor and character were not the same. Lassie is the most high-profile example of the dog actor’s sex (male) not matching the sex of the character (female). Promoting Lassie: The Animal Star and Constructions of “Ideal” American Heroism by Kelly Wolf, from the book Cinematic Canines writes about this in more detail, particularly focusing on how the media and press tried to grapple with a male dog Pal, playing a female dog Lassie. As there was no evidence that the sex of the actor playing the on-screen character impacted its portrayal, we used the sex of the character in our analysis.*

Line 477- comma after 1972

*Authors note: Added, thank you. See page 23, line 469.*

Line 478- comma after 1980s

*Authors note: Added, thank you. See page 23, line 470.*

Line 539- please remove full stop from before the reference

*Authors note: Removed, thank you. See page 26, line 531.*

Line 564-565- please reword as this is unclear

*Authors note: We have reworded this line on page 27, lines 552 - 557 and the passage now reads as:*

*“Armbruster (22)* *expressed that anthropomorphised and talking dogs powerfully confirm our belief in human superiority by finding comedy in how imperfectly they attempt our actions. Instead of helping humans understand and empathise with dogs by making them more human, anthropomorphism may instead be highlighting dogs’ differences from humans. Their imperfection at the things that are supposedly human others them instead of creating a connection.”*

Line 586 and throughout- would purebred dogs sound better than breed dogs?

*Authors note: We used breed dogs throughout to remain consistent with the American Kennel Club’s terminology. See* [*here*](https://www.akc.org/dog-breeds/) *for an example.*

Line 606- would written literature have any effect on dog popularity too?

*Authors note: This is an interesting question, and it seems possible although analysing this would beyond the scope of this study.*
