# Supplementary material for: The making of a (dog) movie star: The effect of the portrayal of dogs in movies on breed registrations in the United States
Source: PLoS One. 2022 Jan 12;17(1):e0261916. doi: 10.1371/journal.pone.0261916 (PMC8754329; doi:10.1371/journal.pone.0261916)
Supplement: Peer review history

# Author comment: Author response to previous submission

**DOI:** 10.1371/journal.pone.0261916.r001

1 Sep 2021

Attachment Submitted filename: Weir_Kessler_2021_ResponseToReviewers.docx Click here for additional data file.

# Peer review: Decision Letter 0

**DOI:** 10.1371/journal.pone.0261916.r002

**Authors:** Simon Clegg

2 Nov 2021

PONE-D-21-28136The making of a (dog) movie star: The effect of the portrayal of dogs in movies on breed registrations in the United StatesPLOS ONE

Dear Dr. Weir,

Thank you for submitting your manuscript to PLOS ONE. After careful consideration, we feel that it has merit but does not fully meet PLOS ONE’s publication criteria as it currently stands. Therefore, we invite you to submit a revised version of the manuscript that addresses the points raised during the review process.

==============================

Many thanks for submitting your manuscript to PLOS One

It was reviewed by two experts in the field, and they have recommended some modifications be made prior to acceptance

I therefore invite you to make these changes and to write a response to reviewers which will expedite revision upon resubmission

I wish you the best of luck with your modifications

Hope you are keeping safe and well in these difficult times

Thanks

Simon

==============================

Please submit your revised manuscript by Dec 17 2021 11:59PM. If you will need more time than this to complete your revisions, please reply to this message or contact the journal office at plosone@plos.org. When you're ready to submit your revision, log on to https://www.editorialmanager.com/pone/ and select the 'Submissions Needing Revision' folder to locate your manuscript file.

Please include the following items when submitting your revised manuscript:

- A rebuttal letter that responds to each point raised by the academic editor and reviewer(s). You should upload this letter as a separate file labeled 'Response to Reviewers'.
- A marked-up copy of your manuscript that highlights changes made to the original version. You should upload this as a separate file labeled 'Revised Manuscript with Track Changes'.
- An unmarked version of your revised paper without tracked changes. You should upload this as a separate file labeled 'Manuscript'.

If you would like to make changes to your financial disclosure, please include your updated statement in your cover letter. Guidelines for resubmitting your figure files are available below the reviewer comments at the end of this letter.

If applicable, we recommend that you deposit your laboratory protocols in protocols.io to enhance the reproducibility of your results. Protocols.io assigns your protocol its own identifier (DOI) so that it can be cited independently in the future. For instructions see: https://journals.plos.org/plosone/s/submission-guidelines#loc-laboratory-protocols. Additionally, PLOS ONE offers an option for publishing peer-reviewed Lab Protocol articles, which describe protocols hosted on protocols.io. Read more information on sharing protocols at https://plos.org/protocols?utm_medium=editorial-email&utm_source=authorletters&utm_campaign=protocols.

We look forward to receiving your revised manuscript.

Kind regards,

Simon Clegg, PhD

Academic Editor

PLOS ONE

Journal Requirements:

When submitting your revision, we need you to address these additional requirements.

1. Please ensure that your manuscript meets PLOS ONE's style requirements, including those for file naming. The PLOS ONE style templates can be found at 

https://journals.plos.org/plosone/s/file?id=wjVg/PLOSOne_formatting_sample_main_body.pdf and 

https://journals.plos.org/plosone/s/file?id=ba62/PLOSOne_formatting_sample_title_authors_affiliations.pdf

Please review your reference list to ensure that it is complete and correct. If you have cited papers that have been retracted, please include the rationale for doing so in the manuscript text, or remove these references and replace them with relevant current references. Any changes to the reference list should be mentioned in the rebuttal letter that accompanies your revised manuscript. If you need to cite a retracted article, indicate the article’s retracted status in the References list and also include a citation and full reference for the retraction notice.

Reviewers' comments:

Reviewer's Responses to Questions

**Comments to the Author**

1. Is the manuscript technically sound, and do the data support the conclusions?

The manuscript must describe a technically sound piece of scientific research with data that supports the conclusions. Experiments must have been conducted rigorously, with appropriate controls, replication, and sample sizes. The conclusions must be drawn appropriately based on the data presented. 

Reviewer #1: Yes

Reviewer #2: Yes

**********

2. Has the statistical analysis been performed appropriately and rigorously? 

Reviewer #1: I Don't Know

Reviewer #2: Yes

**********

3. Have the authors made all data underlying the findings in their manuscript fully available?

The PLOS Data policy requires authors to make all data underlying the findings described in their manuscript fully available without restriction, with rare exception (please refer to the Data Availability Statement in the manuscript PDF file). The data should be provided as part of the manuscript or its supporting information, or deposited to a public repository. For example, in addition to summary statistics, the data points behind means, medians and variance measures should be available. If there are restrictions on publicly sharing data—e.g. participant privacy or use of data from a third party—those must be specified.

Reviewer #1: Yes

Reviewer #2: Yes

**********

4. Is the manuscript presented in an intelligible fashion and written in standard English?

PLOS ONE does not copyedit accepted manuscripts, so the language in submitted articles must be clear, correct, and unambiguous. Any typographical or grammatical errors should be corrected at revision, so please note any specific errors here.

Reviewer #1: Yes

Reviewer #2: Yes

**********

5. Review Comments to the Author

Please use the space provided to explain your answers to the questions above. You may also include additional comments for the author, including concerns about dual publication, research ethics, or publication ethics. (Please upload your review as an attachment if it exceeds 20,000 characters)

Reviewer #1: The manuscript explores an interesting question: while we know that the presence of dog breeds in movies influences their successive popularity, is there an effect of *how* the dogs are portrayed? The manuscript goes in the right way to address the question, but it has in my opinion some issues related to clarity, especially related to the analysis, that need to be addressed before it can be considered for publication. Below I outline the ones I noticed.

Lines 203-204: a transition is missing, in the writing, between how the categories were formed and the hypotheses tested.

Hypotheses: it seems strange that, given the initial question, all the hypotheses predict a positive effect of the categories on breed registrations (when would we expect negative effects?)

Lines 311-321: I would take out this paragraph and simply say that the assumptions were met, pointing the reader to the Appendix. (Also, some parts read strange, such as "The models’ errors were found to be significantly correlated, suggesting the data were time series." )

Hypothesis Testing: It was very difficult for me to figure out the point of the discussion in this section. It needs to be clearly stated at the beginning that they are deciding which of the four categories is more important.

Mostly, why did the authors not use a multilevel model? This would have make possible analysing the effects of all the predictors without choosing a priori the ones that are more important. This is an important point that needs to be addressed by the authors.

Lines 352-359: "Each model with an additional predictor was then tested using an ANOVA to determine if these additions significantly improved the model fit." Wouldn't be AIC more appropriate?

Lines 367-368: I would exclude from the analysis mentions to "trends toward significance".

Results: It makes more sense to present first the main results, and subsequently the results concerning the exploratory analyses, i.e. I would start from the section "Hypothesis Testing".

Lines 449-452: This seems a repetition of what was already said at lines 440-443.

Lines 457-459: These lines are unclear.

Reviewer #2: This is a well written and interesting manuscript which investigates how dogs are portrayed in movies and the effect which this has on their popularity. I only have a few minor comments to make to the authors which is indicative of the quality of the manuscript.

Line 24- I am interested in why you chose the American KC, rather than the UK KC with you based in the UK? Was there a specific reason?

You have a few capital letters dotted throughout it which are not needed, and also sometimes Western is capitalised and not others. Please try to be consistent. Do white and black need capitalising?

Line 61- comma after date

Line 64- comma after methods)

Line 93- full stop needed at the end of the sentence

Line 102- maybe placed on an equal footing?

You have a few bits in bold and underlined, and italicised. Please remove if not needed

Line 168- please reword this as it seems a bit strange

Line 293- comma after paper

Can you please use 0.005 (as an example for the p value. I.e. have a 0 before the decimal point

Line 393- out of interest, do you know the sex of the dog? Could male dogs play a female role and vice versa?

Line 477- comma after 1972

Line 478- comma after 1980s

Line 539- please remove full stop from before the reference

Line 564-565- please reword as this is unclear

Line 586 and throughout- would purebred dogs sound better than breed dogs?

Line 606- would written literature have any effect on dog popularity too?

**********

6. PLOS authors have the option to publish the peer review history of their article (what does this mean?). If published, this will include your full peer review and any attached files.

If you choose “no”, your identity will remain anonymous but your review may still be made public.

**Do you want your identity to be public for this peer review?** For information about this choice, including consent withdrawal, please see our Privacy Policy.

Reviewer #1: No

Reviewer #2: No

[NOTE: If reviewer comments were submitted as an attachment file, they will be attached to this email and accessible via the submission site. Please log into your account, locate the manuscript record, and check for the action link "View Attachments". If this link does not appear, there are no attachment files.]

While revising your submission, please upload your figure files to the Preflight Analysis and Conversion Engine (PACE) digital diagnostic tool, https://pacev2.apexcovantage.com/. PACE helps ensure that figures meet PLOS requirements. To use PACE, you must first register as a user. Registration is free. Then, login and navigate to the UPLOAD tab, where you will find detailed instructions on how to use the tool. If you encounter any issues or have any questions when using PACE, please email PLOS at figures@plos.org. Please note that Supporting Information files do not need this step.

# Author comment: Author response to Decision Letter 0

**DOI:** 10.1371/journal.pone.0261916.r003

30 Nov 2021

Dear PLOS ONE editorial team

We have also attached a response to reviewers file with the responses pasted here.

Sincerely,

Sarah Weir and Sharon Kessler

Comments to the Author

Reviewer #1

The manuscript explores an interesting question: while we know that the presence of dog breeds in movies influences their successive popularity, is there an effect of *how* the dogs are portrayed? The manuscript goes in the right way to address the question, but it has in my opinion some issues related to clarity, especially related to the analysis, that need to be addressed before it can be considered for publication. Below I outline the ones I noticed.

Lines 203-204: a transition is missing, in the writing, between how the categories were formed and the hypotheses tested.

Authors response: Thank you for highlighting this. We have added in a transition on page 10, line 203.

Hypotheses: it seems strange that, given the initial question, all the hypotheses predict a positive effect of the categories on breed registrations (when would we expect negative effects?)

Authors response: Thank you, this is an interesting question. All our hypothesises predict a positive effect because during the literature review we found most, if not all, dogs discussed were depicted as likeable characters. Also, a high proportion of movies that met our inclusion criteria were classed as ‘Family’ or were for children. Therefore, we expected that the portrayals of dogs in movies would generally be positive and would elicit a positive response from moviegoers.

However, we think it would be interesting for future research to better understand what kind of portrayals would have a negative effect on breed registrations. When watching the films, we found that there were some dog breeds depicted as villains (for example the human villain’s Dobermans in Oliver and Company (1988)), however these dogs were not on screen long enough to be included in the present study (were present for less than 5 minutes of screentime). We did have one villain dog, Cujo, however his character was associated with an increase of breed registrations of St. Bernard’s relative to all registrations after the movie. Future studies could create more appropriate coding criteria for villain dogs or perhaps look at other animals kept as pets who are viewed less positively by the public (e.g. snakes).

Lines 311-321: I would take out this paragraph and simply say that the assumptions were met, pointing the reader to the Appendix. (Also, some parts read strange, such as "The models’ errors were found to be significantly correlated, suggesting the data were time series." )

Authors note: We have removed this paragraph and have included the following in the main body on page 15, line 312 – 313, 

“The assumptions of a multiple linear regression model were met, and results of tests are included in Appendix S5.”

Hypothesis Testing: It was very difficult for me to figure out the point of the discussion in this section. It needs to be clearly stated at the beginning that they are deciding which of the four categories is more important.

Authors note: We have rewritten this section and clearly outlined that we are deciding the importance of the predictors and how we calculated this. See page 15, lines 313 - 322.

Following additional suggests from the reviewers (see below), we did run a best subsets analysis, which did not make a piori decisions about which predictors were important, and the results confirmed our initial analysis.

Mostly, why did the authors not use a multilevel model? This would have make possible analysing the effects of all the predictors without choosing a priori the ones that are more important. This is an important point that needs to be addressed by the authors.

Authors note: We ran multiple linear regression because our data is not multilevel with a hierarchical structure. At first appearance our data appears multilevel because dogs seem to be nested in movies. However, we found that dog characters within a movie are not more similar than dog characters between movies. We have graphed this below.

In addition, the presence of remakes or sequels may appear to have the same dog across multiple movies, however these characters are a minority in the data and scored differently in each movie. Of the 48 movies, 6 characters featured in more than 1 movie because of either remakes or sequels. Of these only Rusty in his movies, “Rusty Leads the Way” and “My Dog Rusty” has some of the same scores (Hero = 47% vs 65%, Anthropomorphism = 42% vs 42%, Western Ideals = 22% vs 22% and Nature / Society Boundary = 0% vs 6%). We do not believe that this character would significantly change the results and so we believed that multiple linear regression was the most appropriate method. 

We wanted to avoid overfitting a model with data that did not have a clear multilevel structure and so we used a linear multiple regression.

Lines 352-359: "Each model with an additional predictor was then tested using an ANOVA to determine if these additions significantly improved the model fit." Wouldn't be AIC more appropriate?

Authors note: We chose an ANOVA because we wanted to know which model significantly improved model fit. To ensure using an ANOVA did not bias our results, we calculated the AIC for each of our 4 models and years. The model with the lowest AIC is to be selected and so we were able to compare if the AIC and ANOVA directed us to select the same model. We found that when using the original Hierarchical Stepwise Model Building Approach, AIC results indicated that model 2 which includes Dog Hero and Anthropomorphism was the best fit for the data.

Then, to make sure our a priori approach hadn’t missed a model we hadn’t tested, we then ran the Best Subsets method and used Adjusted R2, CP, BIC (because its more conservative than AIC) to assess which model is the best fit for the data. Again model 2 was found to be the most appropriate (except for 10 year trends where no results were significant). We have added the following to the manuscript on page 19, lines 384 – 389 so it is clear for the reader that the analysis choices did not bias results:

“To confirm the Hierarchical Stepwise a priori approach using an ANOVA to compare models was not biasing the results, AICs were calculated for the models created using a Stepwise model. A Best-Subsets approach was also run to ensure the a prior predictor order was not biasing results, and the resulting models were compared using a adjusted R2, CP’s Mallow and BIC criterion. These approaches confirmed the original findings (See S10 Appendix for results).”

Lines 367-368: I would exclude from the analysis mentions to "trends toward significance".

Authors note: We have removed mentions to “trends toward significance” (see page 17, line 357, page 20, lines 401 - 405 and page 22, lines 452 - 454).

Results: It makes more sense to present first the main results, and subsequently the results concerning the exploratory analyses, i.e. I would start from the section "Hypothesis Testing".

Authors note: We agree and have moved the Characters Analysed section below Hypothesis Testing. We have left the distribution of scores above Hypothesis Testing because it is descriptive data that we believe helps the reader understand the results of the hypotheses.

Lines 449-452: This seems a repetition of what was already said at lines 440-443.

Authors note: We have removed the repetitive information and retained only the information required to understand the figure. The caption on page 22, lines 443 - 446 now reads as

“Female dogs analysed were significantly more likely to have a higher score for Western Ideals. There were 3 male characters who were outliers, all from the One Hundred and One Dalmatians franchise; Pongo from One Hundred and One Dalmatians (1961), Pongo from the 1991 remake and Dipstick from 102 Dalmatians (2000).”

Lines 457-459: These lines are unclear.

Authors note: Thank you for pointing this out. We have changed these lines to the following on page 22 line 448 - 450:

“Wilcox rank sum tests were used to test if dogs were portrayed differently depending on whether the movie was live action or animated. These tests were used because normality tests were significant, p < 0.05”

Reviewer #2

This is a well written and interesting manuscript which investigates how dogs are portrayed in movies and the effect which this has on their popularity. I only have a few minor comments to make to the authors which is indicative of the quality of the manuscript.

Authors note: Thank you!

Line 24- I am interested in why you chose the American KC, rather than the UK KC with you based in the UK? Was there a specific reason?

Authors note: We decided to choose the American KC for a few reasons:

1. Previous research was focused on the US and we wanted to extend this research. We believed using the same registration data would make the results more comparable to existing literature

2. The Kennel Club data was freely available on the AKC digital archive and American dog movies were accessible through streaming platforms

3. Hollywood and American films have historically had a substantial, global cultural impact and so we believed focusing on films that had global reach would create more impactful results. Some of the most popular and influential dog movies of the century were created in the US (Lassie, Rin Tin Tin, Rinty franchises and all of the Disney movies).

It would be interesting for future research to use a similar approach in other countries to understand if this is a global phenomenon or if dogs are portrayed differently in different cultures.

You have a few capital letters dotted throughout it which are not needed, and also sometimes Western is capitalised and not others. Please try to be consistent. Do white and black need capitalising?

Authors note: Thank you for highlighting this. We have capitalised the Western because it is referring to a geographic place and the ideals is now consistently lowercase. However, we have capitalised all names of the hypothesises to make it clear when we are referring to these. We have made this clearer when outlining the hypothesises (page 10, lines 204 - 215). We have gone through and checked the other hypothesises to ensure they meet this rule.

We capitalised Black and White to conform to the APA Style Guidelines which can be found here.

Line 61- comma after date

Authors note: Added, thank you. See page 4, line 61.

Line 64- comma after methods)

Authors note: Added, thank you. See page 4, line 64.

Line 93- full stop needed at the end of the sentence

Authors note: Added, thank you. See page 5, line 93.

Line 102- maybe placed on an equal footing?

Authors note: Added, thank you. See page 6, line 102.

You have a few bits in bold and underlined, and italicised. Please remove if not needed

Authors note: We have removed any stylised elements from the paper. The only remaining words that are italicised are movies names to conform to APA style rules for writing about movies here.

Line 168- please reword this as it seems a bit strange

Authors note: Thank you, we have rewritten this section on page 9, lines 166 - 168 as:

“For example, in John Carpenter’s The Thing (1982), the seemingly friendly husky dog at the beginning of the movie is actually an alien that absorbs everything in its path.”

Line 293- comma after paper

Authors note: Added, thank you. See page 14, line 294.

Can you please use 0.005 (as an example for the p value. I.e. have a 0 before the decimal point

Authors note: We have added a zero before every decimal point throughout the manuscript.

Line 393- out of interest, do you know the sex of the dog? Could male dogs play a female role and vice versa?

Authors note: Yes, we often know the sex of the character and can usually find out the sex of the dog actor playing the role. Sometimes the sex of the dog actor and character were not the same. Lassie is the most high-profile example of the dog actor’s sex (male) not matching the sex of the character (female). Promoting Lassie: The Animal Star and Constructions of “Ideal” American Heroism by Kelly Wolf, from the book Cinematic Canines writes about this in more detail, particularly focusing on how the media and press tried to grapple with a male dog Pal, playing a female dog Lassie. As there was no evidence that the sex of the actor playing the on-screen character impacted its portrayal, we used the sex of the character in our analysis.

Line 477- comma after 1972

Authors note: Added, thank you. See page 23, line 469.

Line 478- comma after 1980s

Authors note: Added, thank you. See page 23, line 470.

Line 539- please remove full stop from before the reference

Authors note: Removed, thank you. See page 26, line 531.

Line 564-565- please reword as this is unclear

Authors note: We have reworded this line on page 27, lines 552 - 557 and the passage now reads as:

“Armbruster (22) expressed that anthropomorphised and talking dogs powerfully confirm our belief in human superiority by finding comedy in how imperfectly they attempt our actions. Instead of helping humans understand and empathise with dogs by making them more human, anthropomorphism may instead be highlighting dogs’ differences from humans. Their imperfection at the things that are supposedly human others them instead of creating a connection.”

Line 586 and throughout- would purebred dogs sound better than breed dogs?

Authors note: We used breed dogs throughout to remain consistent with the American Kennel Club’s terminology. See here for an example.

Line 606- would written literature have any effect on dog popularity too?

Authors note: This is an interesting question, and it seems possible although analysing this would beyond the scope of this study.

Attachment Submitted filename: Weir_Kessler_2021_ResponseToReviewers.docx Click here for additional data file.

# Editor report: Decision Letter 1

**DOI:** 10.1371/journal.pone.0261916.r004

**Authors:** Simon Clegg

14 Dec 2021

The making of a (dog) movie star: The effect of the portrayal of dogs in movies on breed registrations in the United States

PONE-D-21-28136R1

Dear Dr. Weir,

We’re pleased to inform you that your manuscript has been judged scientifically suitable for publication and will be formally accepted for publication once it meets all outstanding technical requirements.

Within one week, you’ll receive an e-mail detailing the required amendments. When these have been addressed, you’ll receive a formal acceptance letter and your manuscript will be scheduled for publication.

An invoice for payment will follow shortly after the formal acceptance. To ensure an efficient process, please log into Editorial Manager at http://www.editorialmanager.com/pone/, click the 'Update My Information' link at the top of the page, and double check that your user information is up-to-date. If you have any billing related questions, please contact our Author Billing department directly at authorbilling@plos.org.

If your institution or institutions have a press office, please notify them about your upcoming paper to help maximize its impact. If they’ll be preparing press materials, please inform our press team as soon as possible -- no later than 48 hours after receiving the formal acceptance. Your manuscript will remain under strict press embargo until 2 pm Eastern Time on the date of publication. For more information, please contact onepress@plos.org.

Kind regards,

Simon Clegg, PhD

Academic Editor

PLOS ONE

Additional Editor Comments:

Many thanks for resubmitting your manuscript to PLOS One

As you have addressed all the comments and the manuscript reads well, I have recommended it for publication

You should hear from the Editorial Office shortly.

It was a pleasure working with you and I wish you the best of luck for your future research

Hope you are keeping safe and well in these difficult times

Thanks

Simon

# Editor report: Acceptance letter

**DOI:** 10.1371/journal.pone.0261916.r005

**Authors:** Simon Clegg

19 Dec 2021

PONE-D-21-28136R1 

The making of a (dog) movie star: The effect of the portrayal of dogs in movies on breed registrations in the United States 

Dear Dr. Weir:

I'm pleased to inform you that your manuscript has been deemed suitable for publication in PLOS ONE. Congratulations! Your manuscript is now with our production department. 

If your institution or institutions have a press office, please let them know about your upcoming paper now to help maximize its impact. If they'll be preparing press materials, please inform our press team within the next 48 hours. Your manuscript will remain under strict press embargo until 2 pm Eastern Time on the date of publication. For more information please contact onepress@plos.org.

If we can help with anything else, please email us at plosone@plos.org. 

Thank you for submitting your work to PLOS ONE and supporting open access. 

Kind regards, 

PLOS ONE Editorial Office Staff

on behalf of

Dr. Simon Clegg 

Academic Editor

PLOS ONE
